# Preferential and Comprehensive Reconstitution of Severely Damaged Sciatic Nerve Using Murine Skeletal Muscle-Derived Multipotent Stem Cells

**DOI:** 10.1371/journal.pone.0091257

**Published:** 2014-03-10

**Authors:** Tetsuro Tamaki, Maki Hirata, Shuichi Soeda, Nobuyuki Nakajima, Kosuke Saito, Kenei Nakazato, Yoshinori Okada, Hiroyuki Hashimoto, Yoshiyasu Uchiyama, Joji Mochida

**Affiliations:** 1 Muscle Physiology & Cell Biology Unit, Tokai University School of Medicine, Kanagawa, Japan; 2 Department of Regenerative Medicine, Division of Basic Clinical Science, Tokai University School of Medicine, Kanagawa, Japan; 3 Department of Urology, Division of Surgery, Tokai University School of Medicine, Kanagawa, Japan; 4 Department of Otolaryngology, Tokai University School of Medicine, Kanagawa, Japan; 5 Department of Respiratory Surgery, Tokai University School of Medicine, Kanagawa, Japan; 6 Teaching & Research Support Center, Tokai University School of Medicine, Kanagawa, Japan; 7 Department of Orthopedics, Division of Surgery, Tokai University School of Medicine, Kanagawa, Japan; University of Minnesota Medical School, United States of America

## Abstract

Loss of vital functions in the somatic motor and sensory nervous systems can be induced by severe peripheral nerve transection with a long gap following trauma. In such cases, autologous nerve grafts have been used as the gold standard, with the expectation of activation and proliferation of graft-concomitant Schwann cells associated with their paracrine effects. However, there are a limited number of suitable sites available for harvesting of nerve autografts due to the unavoidable sacrifice of other healthy functions. To overcome this problem, the potential of skeletal muscle-derived multipotent stem cells (Sk-MSCs) was examined as a novel alternative cell source for peripheral nerve regeneration. Cultured/expanded Sk-MSCs were injected into severely crushed sciatic nerve corresponding to serious neurotmesis. After 4 weeks, engrafted Sk-MSCs preferentially differentiated into not only Schwann cells, but also perineurial/endoneurial cells, and formed myelin sheath and perineurium/endoneurium, encircling the regenerated axons. Increased vascular formation was also observed, leading to a favorable blood supply and waste product excretion. In addition, engrafted cells expressed key neurotrophic and nerve/vascular growth factor mRNAs; thus, endocrine/paracrine effects for the donor/recipient cells were also expected. Interestingly, skeletal myogenic capacity of expanded Sk-MSCs was clearly diminished in peripheral nerve niche. The same differentiation and tissue reconstitution capacity of Sk-MSCs was sufficiently exerted in the long nerve gap bridging the acellular conduit, which facilitated nerve regeneration/reconnection. These effects represent favorable functional recovery in Sk-MSC-treated mice, as demonstrated by good corduroy walking. We also demonstrated that these differentiation characteristics of the Sk-MSCs were comparable to native peripheral nerve-derived cells, whereas the therapeutic capacities were largely superior in Sk-MSCs. Therefore, Sk-MSCs can be a novel/suitable alternative cell source for healthy nerve autografts.

## Introduction

Traumatic peripheral nerve injury can be caused by mechanical trauma such as penetration, crush, traction and lacerations, and may also be associated with long bone fractures, such as those occurring in traffic accidents [1], [2]. Generally, these injuries are classified according to two international standards: the Seddon and the Sunderland [3], [4]. The most serious of these injuries is Seddon’s neurotmesis, equivalent to Sunderland’s fifth degree, which represents complete transection of the nerve, and appropriate surgical treatment is absolutely imperative. For simple transection of the nerve, direct end-to-end suture is possible. In many cases, however, the distance between the proximal and distal stumps is too large to allow direct suture. In such cases, autologous nerve grafts have been used as the gold standard [5], with the expectation of proliferation and activation of nerve graft-associated Schwann cells, which then produce a variety of neurotrophic factors and cytokines, leading to cell adhesion and axonal regeneration [6]. However, the use of autologous nerve grafts for bridging defects requires the sacrifice of healthy nerves, and there are a limited number of suitable sites available for harvesting. Therefore, in the development of alternative sources for nerve autografts, scaffold bridging, which could be of synthetic or biological origin or be resorbable or non-resorbable, has been studied. However, it appears clear that acellular conduits do not facilitate nerve regeneration across long gaps [5].

Acellular conduits are associated with several cell sources, such as Schwann cells and/or Schwann-like cells induced from cultivated bone morrow stromal cells [7], olfactory ensheathing cells [8] and adipose tissue-derived cells [9], and have been investigated as alternatives to autografts, but it is unlikely that these could match or exceed the performance of autografts. Although Schwann cells play a central role in peripheral nerve regeneration, the formation of the endoneurium and/or perineurium by endoneurial fibroblasts and perineurial cells are also important because of their protective role in axons with Schwann cells and the myelin sheath. In particular, the perineurium plays an important role in preventing the passing of large molecules from the epineurium into perineurial fascicles, which is also known as the “blood-nerve-barrier” system [10]. Reconstitution of vascular networks is an inevitable factor in tissue reconstruction. In this regard, cell sources that can give rise to Schwann cells are associated with the capacity to form perineurium/endoneurium, and blood vessels are considered to be the best source for peripheral nerve regeneration.

We previously reported that skeletal muscle interstitium contained multipotent stem cells, as they were Sk-34 (CD34^+^/45^−^) and Sk-DN (CD34^−/^45^−^) cells [11], [12]. These cells were able to differentiate into mesodermal cells (skeletal muscle cells, vascular smooth muscle cells, pericytes and endothelial cells) and ectodermal cells (Schwann cells and perineurial cells) in vivo, and typically exerted synchronized reconstitution of the muscular, vascular and peripheral nervous system in severely damaged skeletal muscle [13]–[15]. This multipotency was confirmed by clonal cell culture and transplantation [11], [12], [15], and the hierarchy of these cells is such that Sk-DN cells are situated upstream of Sk-34 cells in the same lineage [16],[17]. These capacities of skeletal muscle-derived multipotent stem cells (Sk-MSCs) wholly match the demands in peripheral nerve regeneration, except for skeletal myogenesis.

In the present study, we used mixed cell populations isolated from skeletal muscle, which were mainly containing Sk-34and Sk-DN cells, with the aim of assessing their therapeutic utility. We also used the “severe nerve crush injury model” to simulate Seddon’s axonotmesis and/or the Sunderland’s fourth degree, which involves loss of axons, endoneurial tubes, perineurial fasciculi and vascular networks, while continuity of the epineurium is maintained. Therefore, multiple stimulation for peripheral nerves and vascular lineage cells (such as Schwann, endoneurial/perineurial cells and vascular cells) and synergistic effects of transplanted cells are expected. Using this model, we examined the differentiation capacity and contributions of Sk-MSCs in peripheral nerve regeneration. We also applied Sk-MSCs to transected long nerve gap therapy bridging via an acellular conduit. We found that Sk-MSCs differentiated into all of the peripheral nerve support cells, and reconstituted about 94% of transected axons in the long nerve gap, representing the most suitable stem cells for long nerve gap therapy. Experiments using bone marrow stromal cells (BMSCs), sciatic nerve-derived cells (SNDCs) and healthy nerve grafts were also performed as comparative studies.

## Results

### Evaluation of Nerve Crush Injury Model

**Figure 1:**
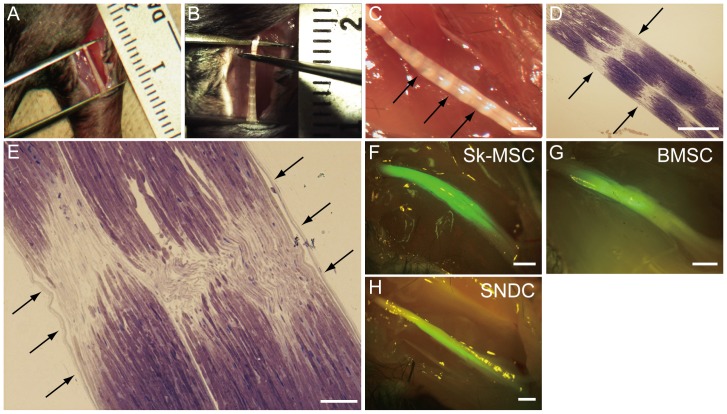
Evaluation of nerve crush injury model, and macroscopic observations at 4 weeks after transplantation. (A) Determination of crush distance and (B) nerve crush by forceps and (C) features immediately after crush damage under stereomicroscope. Nerve length was strictly determined by forceps fixed at 7-mm distance (A), and crush damage was added using other forceps from the vertical direction to the nerve (B). Translucent bands, which are evidence of nerve crush damage, were clearly evident immediately after surgery (C). (D and E) To confirm the details of the present nerve damage model, 10-minute post-damage nerve was prepared as a resin-section, and was stained with toluidine-blue. Several complete disruptions of nerve fiber bundles could be seen in the longitudinal section (arrows in D), corresponding to the translucent bands in (C). However, continuous epineurium (an envelope of entire nerve) was maintained (arrows in E). (F–H) GFP^+^ tissues were also detectable under the stereomicroscope at 4 weeks after transplantation. Stronger and widespread emission was observed with Sk-MSC transplantation. Scale bars represent 1 mm (C, F–H), 500 µm (D) and 200 µm (E).

The validity of the present severe nerve crush injury model was first evaluated in detail (**Fig.** **1**). We applied crush damage along a 7-mm section using forceps (**Fig. 1A, B**) and confirmed several intermittent and complete disruptions of nerve fibers (**Fig. 1C, D**), whereas continuous epineurium (envelope surrounding the entire nerve) was evident in this crush model (arrows in **Fig. 1E**), meeting the criteria for Sunderland’s fourth degree. At 4 weeks after transplantation, engrafted Sk-MSCs, BMSCs and SNDCs were detectable as GFP^+^ tissues under fluorescence stereomicroscopy. Stronger and widespread emissions could be seen after Sk-MSC transplantation than in BMSC and SNDC, with the expectation of greater engraftment ability as a constant trend throughout the experiment (**Fig. 1F–H**).

In the present study, we performed 7 types of transplantation in the sciatic nerve crush injury model; 3 types of Sk-MSC (freshly isolated, and -3d- and -7d cultured), 2 types of BMSC (freshly isolated and -7d cultured) and 2 types of SNDC (freshly isolated from intact and 4-day post-damaged nerves). The results of successful engraftments and total number of animal usage are summarized in **Table 1**. Typically, unsuccessful engraftments were observed with freshly isolated BMSC and fresh-intact SNDC. These results correspond to the previously reported impaired nerve regeneration by undifferentiated BMSCs [18], [19], and also suggest that the regeneration capacity of non-stimulated cells in the sciatic nerve is impaired. Therefore, these two groups were subsequently excluded. In addition, unexpected cell fusion following Sk-MSCs transplantation was not fully detected in the confirmation experiment using the Cre-loxP system.

**Table 1:** Summary of transplanted cells into nerve crush injury model, and engraftment results.

Cell type	Condition (the number of animal usage)	Result
Skeletal muscle-derived multipotent stem cells(Sk-MSC)	Freshly isolated (n = 41)	Success
	3 day cultured (Sk-MSC-3d) (n = 73)	Success
	7 day cultured (Sk-MSC-7d) (n = 59)	Success
Bone marrow stromal cells (BMSC)	Freshly isolated (n = 5)	Non-success
	7 day cultured (BMSC-7d) (n = 16)	Success
Sciatic nerve-derived cells (SNDC)	Freshly isolated from non-damaged (intact) nerve (n = 5)	Non-success
	Freshly isolated from damaged (crashed) nerve (n = 11)	Success
Cre-loxp mice (Sk-MSC)	3 day cultured (Sk-MSC-3d) (n = 10, one for donor and 4 for recipientswith respect to one another)	Non-success

Successful engraftments were determined by the fluorescence stereomicroscope and with the following histological analysis. As results, successful engraftments could be obtained in all three types of Sk-MSC (fleshly isolated, 3d cultured, and 7d cultured), 7d cultured BMSC, and SNDC isolated from 4d post-damaged sciatic nerve. Note that sufficient expansion of the BMSC could not obtained within 3 days, and SNDC was not able to culture under normal plane condition as used in the Sk-MSC and BMSC, thus, there were excluded from the experimental setting.

### Cellular Engraftment and Quantitative Analysis of Regenerated Axons, Myelin and Blood Vessels in Sk-MSC, BMSC and SNDC Transplanted Nerves

**Figure 2:**
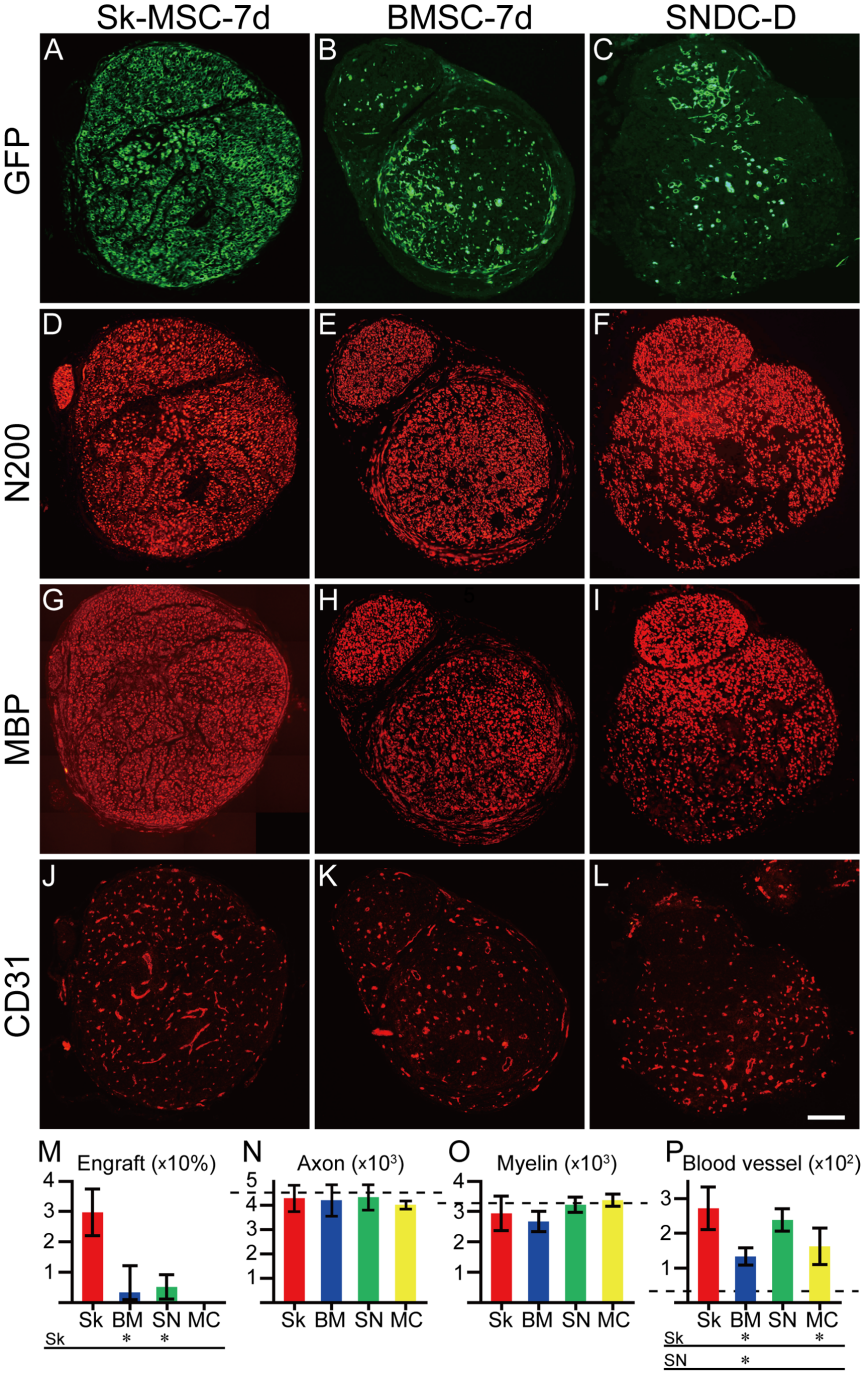
Cellular engraftment and comparison of regenerated axons, myelin and blood vessels in damaged portion of Sk-MSC-7d-, BMSC-7d- and SNDC-D-transplanted nerves at 4 weeks after injection. Comparisons were performed among 7-day cultured Sk-MSCs (Sk-MSC-7d) and BMSCs (BMSC-7d), freshly isolated SNDCs from damaged sciatic nerve (SNDC-D), and medium control (MC) groups based on the results shown in Fig. 1. (F–H). (A–C) Typical engraftment on whole cross-sections of each transplantation group (except for MC group). (D–F) Typical staining of regenerated axons as N200^+^, and myelin as MBP^+^ (G–I) and blood vessel formation as CD31^+^ regions (J–L). (M–P) Comparison of the above factors (Sk = Sk-MSC-7d, BM = BMSC-7d, SN = SNDC-D and MC = medium control). (M) Percentage of mean GFP^+^ area/total area on whole cross-sections, as compared to relative engraftment ratio. (N) Mean number of axons. (O) Mean number of myelin signals. (P) Mean number of blood vessels. Dotted lines in (N, O and P) indicate the mean number of axons, myelin signals and blood vessels in the corresponding portion of normal sciatic nerve (4625±470, 3179±760 and 27±4, respectively). Significantly greater cellular engraftment ratio and blood vessel formation was evident in the Sk-MSC group. N-200; Neurofilament 200, MBP; Myelin basic protein. *P≤0.05; all scale bars represent 200 µm.

In order to evaluate the contributions of transplanted cells to damaged nerve recovery, relative engraftment ratio and number of regenerated axons, myelin and blood vessels in the damaged portion was counted in each cross-section (**Fig. 2**). Comparisons were performed among Sk-MSC-7d (n = 9) and BMSC-7d (n = 7), SNDC-D (n = 6) and medium control (MC, n = 5) groups based on the values in intact controls (n = 5, dotted lines in **Fig. 2M–P**). Sk-MSC-7d showed a significantly greater engraftment ratio when compared to the BMSC-7d and SNDC-D groups, and the values in latter two groups were similar (see **Fig. 2A–C, M**). The number of axons and myelin signals were similar in all four groups (**Fig. 2N, O**), and were largely the same as the levels seen in intact controls, thus suggesting natural/spontaneous recovery occurs within 4 weeks. Therefore, donor cell engraftments were considered to work together with recipient cells during natural healing. Importantly, all four groups showed a significantly higher number of blood vessels than intact controls in the following order: Sk-MSC>DNDC-D>BMSC-7d>MC, with the values of Sk-MSC-7d and SNDC-D being significantly greater when compared with the other two groups (**Fig. 2P**). Taken together, it is likely that natural/spontaneous nerve regeneration is basically induced following increases in the number of blood vessels within the 4-wk recovery period in the present crush injury model, and this trend was most prominent in the Sk-MSC group. Increased blood vessel formation may contribute sufficient oxygen and nutrition supply, as well as removal of waste products, and lead to faster recovery of damaged nerve fibers.

### Cellular Differentiation of Sk-MSCs, BMSCs and SNDCs in Damaged Nerve Niche

**Figure 3:**
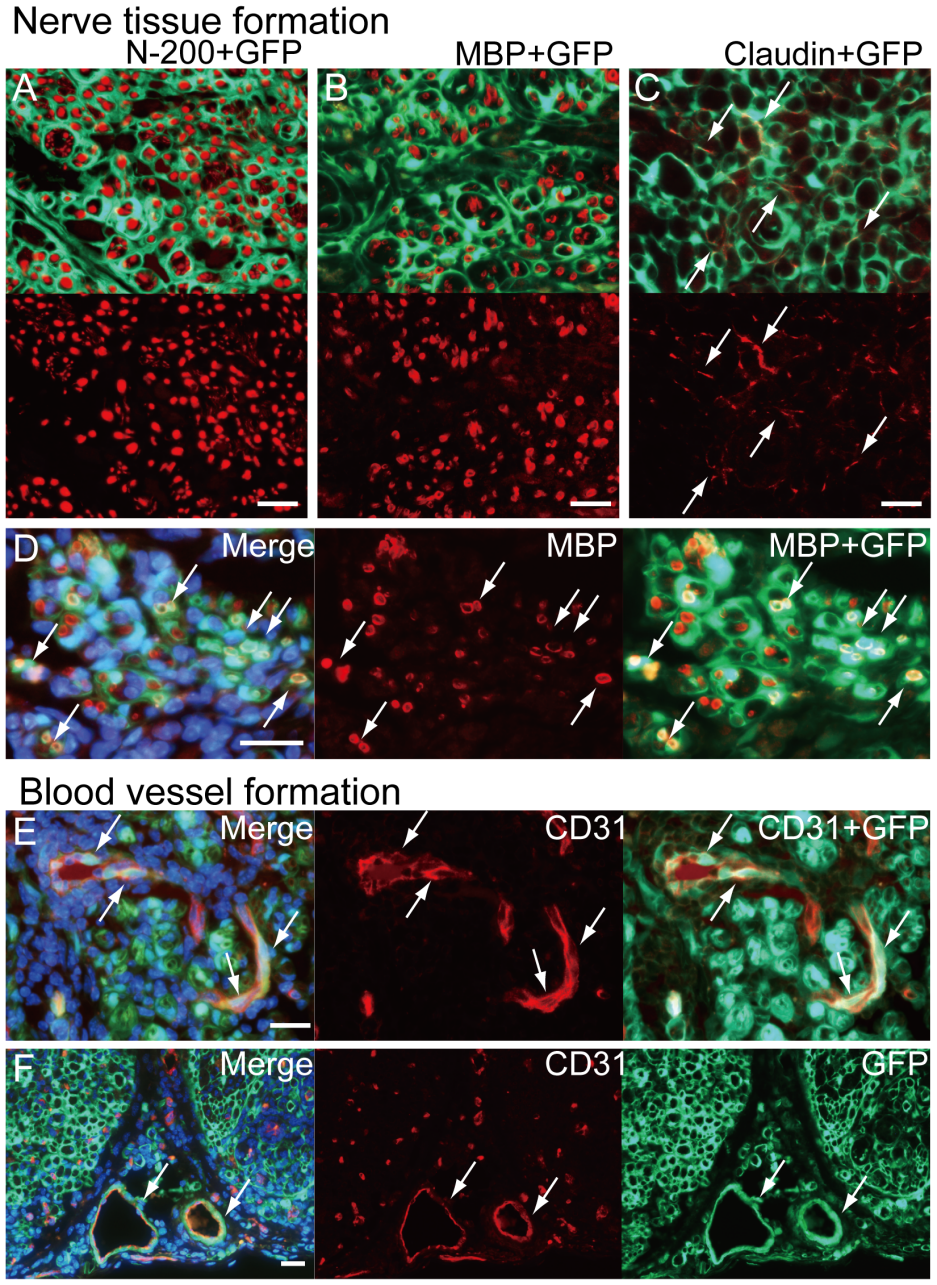
Cellular differentiation of Sk-MSCs in damaged nerve niche at 4 weeks after injection. (A) Tissues having strong GFP emission actively encircled single or multiple axons stained by N-200, thus suggesting formation of perineurium and endoneurium in the Sk-MSC-7d group. This was a common trend in the Sk-MSC-transplanted groups throughout the experiment. (B) GFP^+^ circles (probably perineurium/endoneurium) also enclosed single/multiple myelin sheaths stained by MBP. (D) Some showed double positive staining for BMP and GFP, thus suggesting donor cell-derived myelin formation (arrows in D). (C) Several claudin^+^ reactions were located on donor-derived GFP^+^ perineurium (arrows in C), demonstrating the formation of tight-junctions. (E) Incorporation of GFP^+^/CD31^+^ cells into blood vessels inside the nerve bundle (arrows in E). Incorporation was seen frequently, but not in all cases. (F) Similar contributions were observed in the large conduit blood vessels located outside of nerve bundles (arrows in F), and GFP^+^ donor cell contributions extended from the tunica media to the adventitia in this case. All scale bars represent 50 µm.

**Figure 4:**
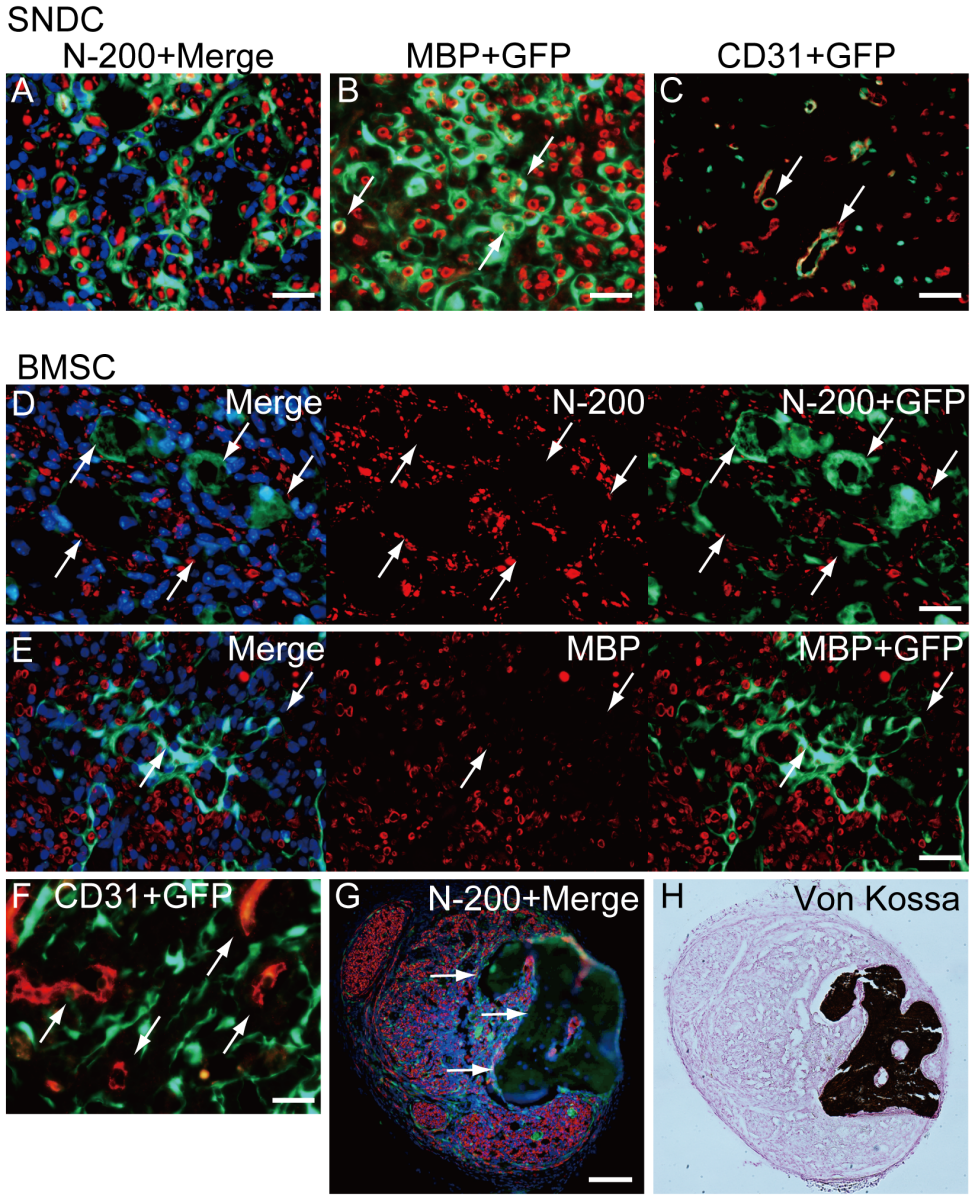
Cellular differentiation of SNDC and BMSC in damaged nerve niche at 4 weeks after injection. (A–C) Typical differentiation of transplanted damaged sciatic nerve-derived freshly isolated cells (SNDC-D). SNDC-D showed differentiation into perineurial/endoneurial cells that encircled the axon and myelin (A), myelin-forming Schwann cells showed double-positive staining for MBP and GFP (B), and endothelial cells (CD31^+^/GFP^+^) were incorporated into blood vessels (C). These differentiation potentials correspond to Sk-MSC-7d (see Fig. 3), whereas the relative engraftment capacity was significantly lower in SMDC-D (see Fig. 2M). (D–H) Typical behavior of transplanted BMSC-7d. Definitive perineurium/endoneurium formation closely encircling axons and myelin, which were observed in both Sk-MSCs and SNDCs, was not seen in the BMSC-7d group; instead, axons with very small diameter and weak reactions for MBP were prominent (D, E). In addition, hollow cavity-like structures around engrafted GFP^+^ tissues (cells) were frequently observed (arrows in D, E). This trend was consistently observed throughout BMSC-7d transplantation. Similarly, GFP^+^ cells (tissues) were not incorporated into CD31^+^ blood vessels (arrows in F). Interestingly, some osteogenesis was observed with BMSC transplantation (G, H), but only in one case (1/13). A large dark area that superficially resembled a large hollow cavity under fluorescence immunohistochemistry (arrows in G) was positive for von Kossa staining (H), representing calcified bone formation in the damaged peripheral nerve niche. Scale bars represent 50 µm (A–F), 200 µm (G).

At 4 week after transplantation, transplanted sciatic nerves in the three groups in **Fig. 2** were histologically analyzed by higher magnifications (**Fig. 3** and **4**). Vigorous formation of GFP^+^ perineurium/endoneurium encircling single and/or multiple axons and myelin were evident as typical characteristics of Sk-MSC-7d transplantation (**Fig. 3A, B**). Several claudin^+^ reactions were located on GFP^+^ perineurium showing formation of tight junctions [20], [21] by donor-derived cells (**Fig. 3C**). Double positive reactions for GFP/MBP were also observed as proof of myelin formation by donor-derived Schwann cells (**Fig. 3D**). In addition, incorporation of donor-derived GFP^+^ endothelial (CD31^+^) cells with blood vessels inside of nerve bundle (**Fig. 3E**) were frequently observed (but not all cases). Similar contributions were also observed in the large conduit blood vessels located outside the nerve bundle (**Fig. 3F**), and GFP^+^ tissues extended from the tunica media to the adventitia in this case. These multiple contributions for the vascular system correspond to the original reported function of Sk-MSCs exerted in damaged skeletal muscle niche [**13]**–**[15]**. Thus, transplanted donor-derived Sk-MSC-7d actively differentiated into Schwann and perineurial/endoneurial cells, in addition to vascular-related cells and contributed to nerve and vascular reconstitution. All of these trends were consistently observed in Sk-MSC-3d (described below) and Sk-MSC-7d transplantation.

After SNDC-D transplantation, the same characteristics of the above Sk-MSC-7d were observed (**Fig. 4A–C**). However, the expansion was apparently smaller than with Sk-MSC-7d as a result of the significantly lower engraftment ratio in SNDC-D (see **Fig. 2M**). Therefore, the differentiation capacities of Sk-MSCs and SNDCs were basically the same in the damaged peripheral nerve niche, but the engraftment capacity was significantly higher in Sk-MSCs.

After BMSC-7d transplantation, the trend was apparently different from the other two groups **(** **Fig. 4D–F**). There was no definitive formation of perineurium/endoneurium or incorporation into CD31^+^ blood vessels. Instead, axons with very small diameter and thin/weak reactions for MBP were prominent. In addition, hollow cavity-like structures around engrafted GFP^+^ tissues (cells) were frequently observed. Interestingly, some osteogenesis was observed in one case (**Fig. 4G, H**).

**Figure 5:**
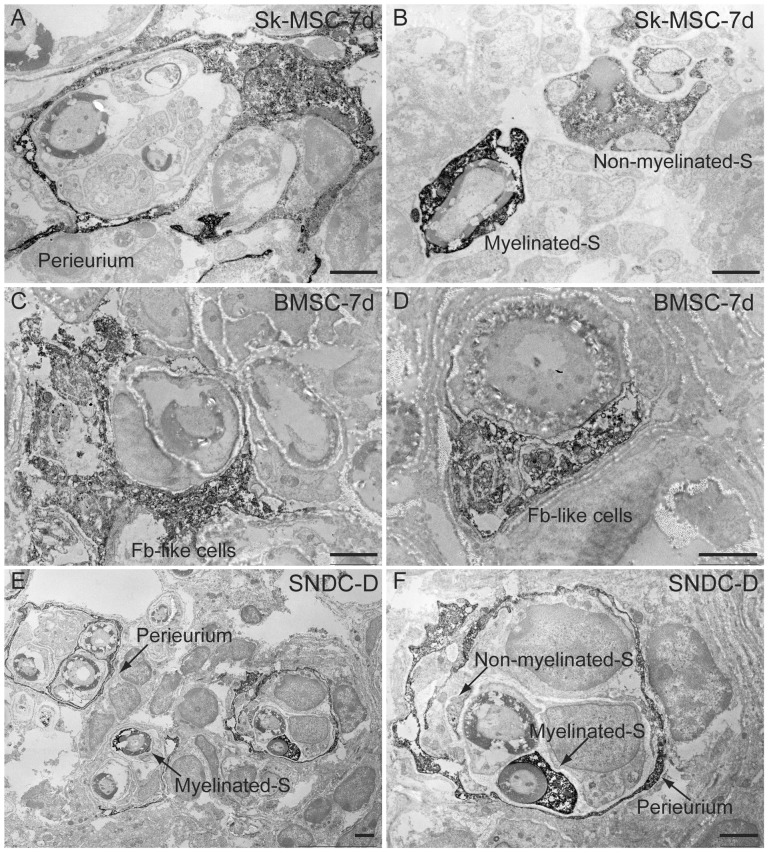
Detailed analysis of engrafted cell differentiation into peripheral nerve tissues in damaged sciatic nerve by immunoelectron microscopy at 4 week. Three mice/group were used in this analysis. Anti-GFP antibody was used and positive reactions are represented as black dots. (A, B) Confirmation of Sk-MSC-7d differentiation into Schwann cells and formation of perineurium. (E–F) Similar confirmation of SNDC-D differentiation into Schwann cells and formation of perineurium. (C–D) Localization of BMSC-7d-derived fibroblast-like structure cells between Schwann cells and perineurium. Note that there were no other specific characteristics observed in BMSC-7d transplantation. S = Schwann cell. Fb = Fibroblast. Scale bars represent 2 µm.

The differentiation of engrafted Sk-MSC-7d and SNDC-D into Schwann cells, as well as the formation of perineurium, was also strictly assessed by immunoelectron microscopy (**Fig. 5A–B and E–F**). However, after BMSC-7d transplantation, fibroblast-like structures located between Schwann cells and perineuriums with no other assertive characteristics were detected (**Fig. 5C–D**). Therefore, the cause-and-effect relationship of BMSC transplantation was unclear in the present study, in contrast to other Sk-MSC-7d and SNDC-D.

### Expression of mRNAs Specific to Peripheral Nerve, Vascular and Skeletal Muscle Lineages Before and After Transplantation

**Figure 6:**
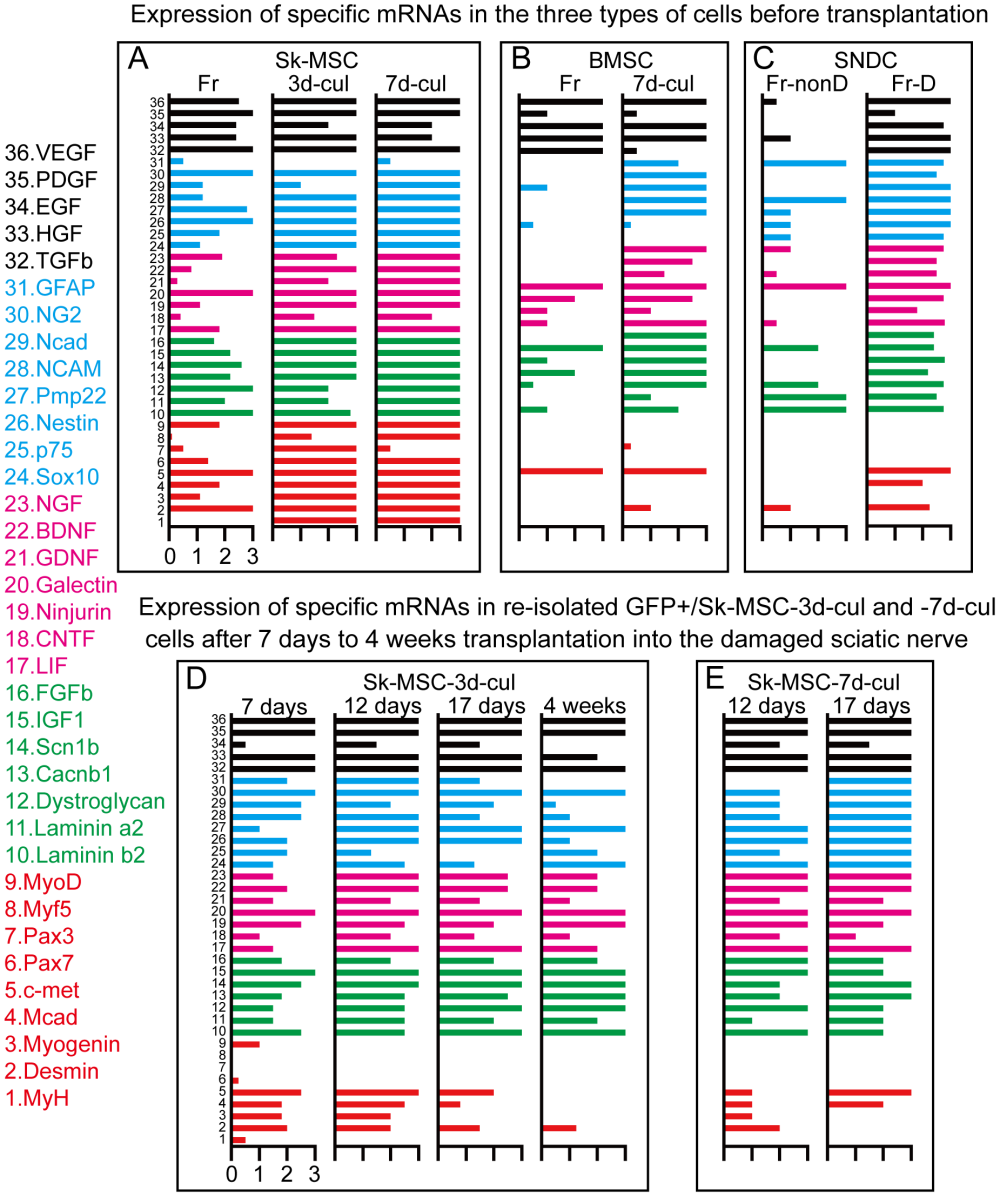
Expression of specific mRNAs for peripheral nerves, vascular and skeletal muscle lineages in three types of cells before transplantation, and in re-isolated Sk-MSC-3d and -7d cells after transplantation into damaged sciatic nerve. Thirty-six primers were used in this analysis. Blue bars represent differentiation markers for peripheral nerve cells. Pink bars represent nerve growth and neurotrophic factors. Red bars are differentiation markers for skeletal muscle, and green bars are common factors. Full names, details and roles of each primer used in this analysis are summarized in Table S1. (A–C) Upper panels show expression patterns in the three types of cells before and after expansion culture (before transplantation); Sk-MSCs (A), BMSCs (B) and SNDCs (C). Freshly isolated Sk-MSCs (Fr) expressed all markers in the above 4 categories, and this expression was consistently enhanced during expansion cell culture (-3d and -7d in A), except for GFAP (mature Schwann cell marker naturally expressed in intact nerve, blue bar No. 31). In freshly isolated BMSCs (Fr), relatively strong expression of vascular-related growth factors (black) was observed, but the other three categories showed weak expression (B). After 7 days of expansion culture (-7d), expression of nerve, vascular and common factors were enhanced, but no enhancement of skeletal muscle markers was seen (black bars in B). There were no assertive characteristics in freshly isolated SNDCs from intact sciatic nerve (Fr-nonD in C), but uniform and strong expression of factors other than skeletal muscle lineage were observed in freshly isolated SNDCs from 4 days after damage (Fr-D in C). (D–E) Lower panels show expression in Sk-MSC-3d and -7d after transplantation into damaged sciatic nerve niche. Engrafted Sk-MSC-3d and -7d were enzymatically re-isolated from regenerating sciatic nerve at 7, 12, 17 days and 4 weeks after transplantation, and were sorted as GFP^+^ cells and subjected to mRNA analysis (D, E). Strong expression of skeletal myogenic mRNAs, which was observed in -3d and -7d cultured Sk-MSC preparations (red bars No. 1–9 in A) were gradually diminished with time after transplantation (compare A to D, E), but these decreases were faster in 7d-cul than in 3d-cul (compare at 12 and 17 days after transplantation in D and E), thus suggesting that myogenic potential was reduced after longer culture periods. However, expression of the remaining 3 categories (No. 10–36) was consistently/continuously observed in both preparations (D, E). Expression patterns of Sk-MSC-7d after 17 days closely resemble to those in SNDC from damaged nerve (Fr-D in c vs. 17 days in E).

Relative expression of specific mRNAs in Sk-MSCs was compared to that in native SNDCs before and after transplantation. Thirty-six markers were selected in relation to cellular commitment, growth and regeneration of peripheral nerves, blood vessels, skeletal muscle and common factors (**Fig. 6** **, and Table S1**).

No clear expression was detected in freshly isolated SNDCs from intact sciatic nerve; thus, this was considered to be the normal state (**Fig. 6C**). However, uniform and strong expression of three categories other than skeletal muscle lineage were observed in freshly isolated SNDC from 4 days after damage, and was considered to be the regenerative response of native peripheral nerve tissue (**Fig. 6C**). Based on these responses, freshly isolated Sk-MSCs showed expression in the above 4 categories, and this was consistently enhanced to maximum during expansion culture (-3d to -7d in **Fig. 6A**), except for GFAP. This clearly indicates that Sk-MSCs are suitable for nerve regeneration rescue, while skeletal myogenic marker expression is strong. As a comparative control, freshly isolated BMSCs showed relatively strong expression of vascular-related factors, but the remaining factors were enhanced after 7 days of culture, except for skeletal muscle factors and Sox10 (**Fig. 6B**). In this regard, it was likely that preliminary expression of specific mRNAs and actual cell differentiation after transplantation did not always correspond, as was observed in BMSC-7d. Importantly, the supportive paracrine effects of these transplanted cells were indicated by the increased number of vascular supplies in the damaged nerve (**Fig. 2P**).

We also analyzed expression in re-isolated Sk-MSC-3d and -7d at 7, 12, 17 days and 4 weeks after transplantation in order to follow differentiation during nerve regeneration (**Fig. 6D, E**). Strong expression of skeletal myogenic mRNAs, which was observed before transplantation, diminished gradually after transplantation (**Fig. 6A** vs. **D, E**). These decreases were relatively fast at Sk-MSC-7d, as compared to -3d cultured cells (**Fig. 6D** vs. **E**). Therefore, longer culture periods may induce preferential attenuation of the myogenic potential of Sk-MSCs after transplantation, while expression of the remaining 3 categories was consistently/continuously maintained in both -3d and -7d preparations. In addition, the pattern of Sk-MSC-7d at 17 days closely resembled that of SNDC-D (**Fig. 6C** vs. **E**). These results suggest that ideal milieu-dependent cell differentiations and paracrine effects occur in Sk-MSCs following transplantation. Five mice were used for analysis at each stage in both groups.

### In Vivo Differentiation Potential of Re-isolated Sk-MSC-3d After “2^nd^ Transplantation” Into Damaged Skeletal Muscle and Sciatic Nerve

**Figure 7:**
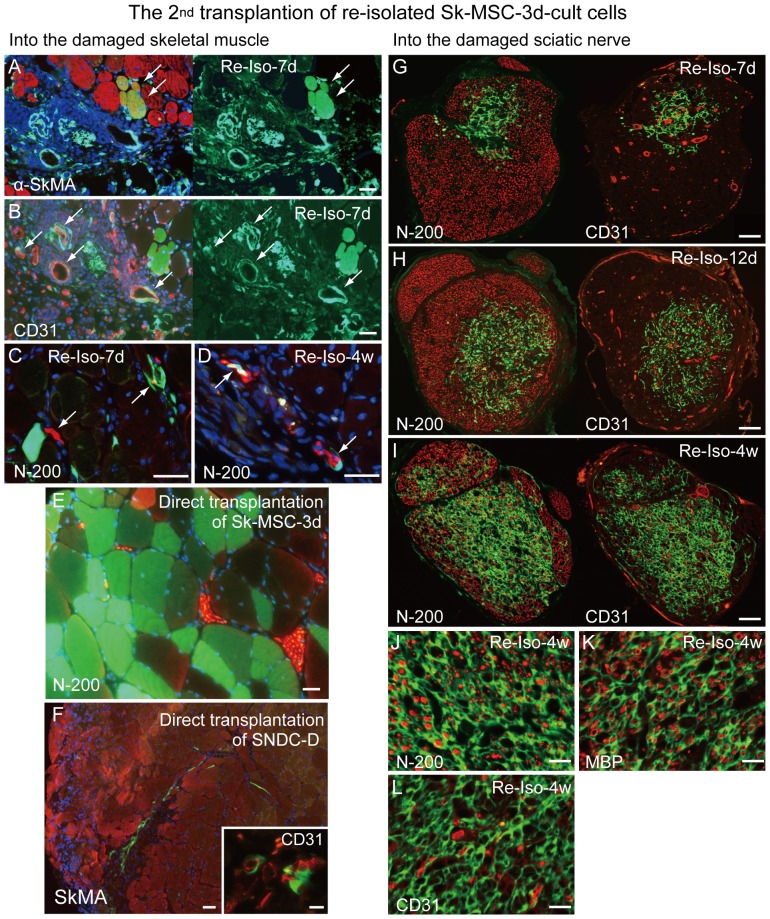
In vivo differentiation potential of re-isolated Sk-MSC-3d after “2^nd^ transplantation” into damaged skeletal muscle and sciatic nerve. To confirm the differentiation potential of once engrafted Sk-MSC-3d, GFP^+^ cells were re-isolated from transplanted crushed nerves (using same method as described in previous RT-PCR analysis) from 1^st^ transplanted mice (n = 10), and re-transplanted into both the damaged skeletal muscle and sciatic nerve models (2^nd^ transplantation). Re-isolation and 2^nd^ transplantation were performed at 7, 12, 17 days and 4 weeks after 1^st^ transplantation (n = 3 in each stages in both the damaged skeletal muscle and sciatic nerve), and final sampling was performed at 4 weeks after 2^nd^ transplantation. The left column shows transplantation into damaged skeletal muscle, and the right column shows transplantation into damaged sciatic nerve. (A) Some GFP^+^ muscle fibers were detected in limited areas (arrows in A). (B, C) Incorporation of GFP^+^ cells to the vascular (arrows in B) and peripheral nerve (arrows in C) tissues were also evident. There were no GFP^+^ muscle fibers detected thereafter, with a lower rate of cellular engraftment (at 12 and 17 days, data not shown). (D) Few GFP^+^ cells related to nerves were seen in the case of re-isolated-4w (arrows in D). (E) There was a large number of GFP^+^ muscle fibers near the N-200^+^ nerve bundles when Sk-MSC-3d were directly transplanted into damaged muscle (as 1^st^ transplantation), thus indicating vigorous skeletal myogenic potential. These data indicate that diminished engraftment capacity of Sk-MSC-3d is associated with reduced myogenic potential in the 2^nd^ transplantation. (F) When original SNDC-D was directly transplanted into damaged skeletal muscle niche, they were unable to differentiate into muscle fibers, and their engraftment capacity was quite low. (G–I) 2^nd^ transplantation of re-isolated Sk-MSC-3d cells at -7d (G), -12d (H) and -4 w (I) into damaged sciatic nerve. Number of engrafted cells increased gradually with time after 2^nd^ transplantation. (J–L) Similar trends in cell differentiation which was observed in 1^st^ transplantation were seen for 2^nd^ transplantation (refer to Figs. 2 and 3). Scale bars represent 50 µm (A–F, and J–L), 20 µm (inset of F) and 200 µm (G–I).

In order to further confirm the results of previous RT-PCR analysis (**Fig. 6**), re-isolated Sk-MSC-3d were re-transplanted (hereinafter, 2^nd^ transplantation) into the damage skeletal muscle and sciatic nerve to analyze the retention differentiation potential in vivo (**Fig. 7**).

In damaged skeletal muscle, when Sk-MSC-3d were directly transplanted into the damaged muscle (as the 1^st^ transplantation), there was a large number of GFP^+^ muscle fibers near the N-200^+^ nerve bundles (**Fig. 7E**), representing vigorous skeletal myogenesis. However, in the 2^nd^ transplantation of 7d-re-isolated Sk-MSC-3d, few GFP^+^ muscle fibers were detected, whereas contributions to vascular and peripheral nerve tissues were observed (**Fig. 7A–C**). There was no donor cell-derived muscle fiber formation in the rest of the re-isolated cells thereafter, and engraftment capacity itself also apparently reduced in the case of 4wk-re-isolated cells (**Fig. 7D** **)**. This was similar to the case when original SNDC-D were directly transplanted into damaged skeletal muscle niche (**Fig. 7F**). These results supported the above RT-PCR data (**Fig. 6**). It is therefore likely that the capacity of Sk-MSCs in skeletal muscle niche is reduced after the 1^st^ transplantation into the damaged sciatic nerve.

In contrast, the reverse trend was observed after the 2^nd^ transplantation into damaged sciatic nerve niche, as diminished engraftment capacity was observed for 7d re-isolated Sk-MSC-3d (**Fig. 7G**). This subsequently recovered gradually (**Fig. 7H**) and vigorous engraftment was observed in the 4wk-re-isolated cells (**Fig. 7I**). The trends for engrafted re-isolated Sk-MSC-3d (**Fig. 7J–L**) were similar to those for the 1^st^ transplantation (see **Figs. 2** and **3**) and SNDC-D transplantation (**Fig. 4A–C**). Thus, cellular instability of Sk-MSCs is apparently induced in the first 2 weeks after transplantation, and after losing their original myogenic differentiation capacity, suspended cells may be discarded. However, cellular adaptation of transplanted Sk-MSCs progresses gradually and is largely accomplished within 4 weeks, resulting in activated peripheral nerve cells such as in SNDC-D. Note, the engraftment capacity of re-isolated Sk-MSCs was apparently higher than that of the original SNDC-D (compare **Fig. 7I** vs. **Fig. 4A–C**), but the reasons are unclear.

### Disappearance of Skeletal Myogenic Cells in Damaged Sciatic Nerve

**Figure 8:**
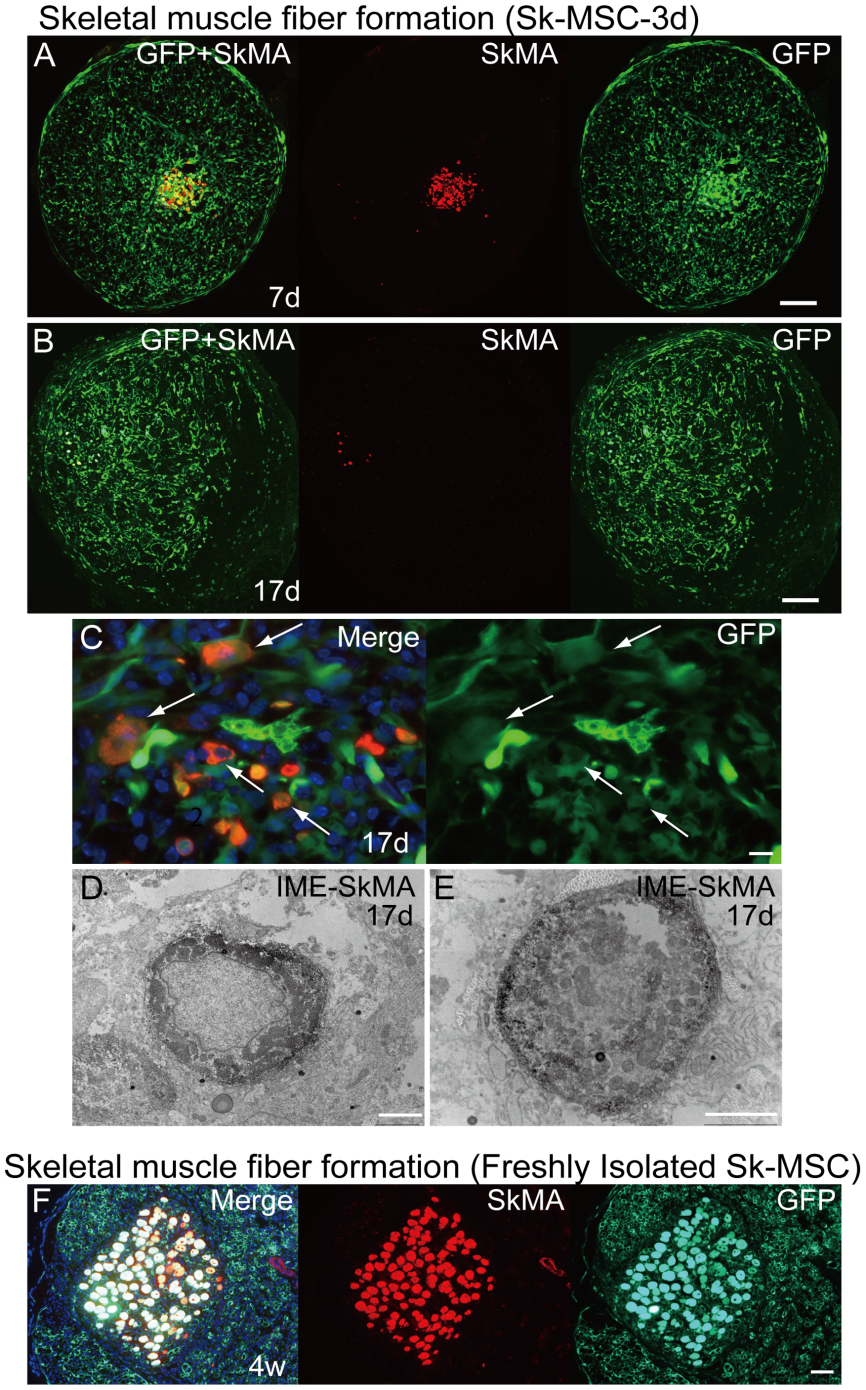
Disappearance of skeletal myogenic cells in damaged sciatic nerve. Data are presented as (A) 7 days, (B–E) 17 days and (F) 4 weeks after Sk-MSC-3d transplantation. (A) SkMA^+^ cells were observed in the damaged sciatic nerve at 7 days after transplantation and were typically aggregated. These cells typically showed strong SkMA^+^ reactions at this time point. However, the number of SkMA^+^ cells was markedly lower at 17 days after transplantation (B), and reactions for anti-SkMA and emission of GFP were both weaker (arrows in C). (D, E) Immunoelectron microscopy of SkMA^+^ cells. The same antibody, which was used for immunohistochemistry, was used and reactions against DAB were represented as black dots. Typical immature features of myogenic cells, such as central nuclei, fewer myofibrils and low nuclear-cytoplasmic ratio, were evident at 17 days after transplantation. This may be representative of the degenerative phase of myotubes, as more intense reactions of SkMA were detectable at 7 days, and there were no SkMA^+^ cells at 4 weeks after transplantation. (F) In contrast, freshly isolated non-cultured Sk-MSCs showed vigorous myogenic potential, and these fibers did not disappear, even at 4 weeks after transplantation. These results suggest that the strong myogenic potential in the freshly isolated Sk-MSCs was not affected by the crushed nerve niche, and muscle fiber formation could be established. We also performed the same analysis for Sk-MSC-7d, and compared the residual ratio of SkMA^+^ cells at 17 days after transplantation among three groups. For Sk-MSC-3d, SkMA^+^ cells remained in 50% of cases (9/18), while -7d showed 33% (7/21), but all SkMA^+^ cells disappeared at 4 weeks after transplantation, thus, residual ratio was 0%. However, freshly isolated Sk-MSCs showed a 100% residual ratio (8/8), and this was maintained even after 4 weeks (not disappeared, thus, 100% residual ratio). SkMA = skeletal muscle actin. Scale bars represent 200 µm (A, B), 10 µm (C), 2 µm (D, E) and 50 µm (F).

In order to follow up the results in **Fig. 6** and **Fig. 7**, disappearance of skeletal myogenesis in Sk-MSC-3d in damaged nerve niche was successively analyzed from 7 days to 4 weeks after transplantation using immunohistochemistry and immunoelectron microscopy (**Fig. 8**). The results clearly indicated that SkMA^+^ cells were present at 7 days after transplantation (**Fig. 8A**). At 17 days, however, the number of SkMA^+^ cells decreased markedly, with weak reactions for anti-SkMA and attenuation of GFP emission observed (**Fig. 8B, C**), probably due to the degenerative phase of myogenic cells. Degenerative features were also confirmed by immunoelectron microscopy at 17 days after transplantation, including central nuclei with fewer myofibrils, even after sufficient time for differentiation (**Fig. 8D, E**). Interestingly, when freshly isolated Sk-MSCs were transplanted into the damaged nerve, there were numerous cells that were strongly positive for SkMA, even at 4 weeks after injection (**Fig. 5E**). This suggests that the myogenic potential of freshly isolated Sk-MSCs was too strong to be eliminated by the damaged nerve niche. We also performed the same analysis for Sk-MSC-7d, and compared the residual ratio of SkMA^+^ cells at 17 days after transplantation among the three groups. The results were as follows: freshly isolated (100%, 8/8)>-3d culture (50%, 9/18)>-7d culture (33%, 7/21). SkMA^+^ cells remained present even after 4 weeks in the case of freshly isolated cells, but had disappeared completely in the other two groups. Based on the results in **Fig. 6** **,** **7** and **8**, it was clear that the myogenic potential of Sk-MSC in vivo is affected by culture conditions before transplantation; thus, the myogenic potential of Sk-MSC is controllable before transplantation.

### Characterization of Sk-MSC-3d and SNDC-D Before Transplantation by FACS

**Figure 9:**
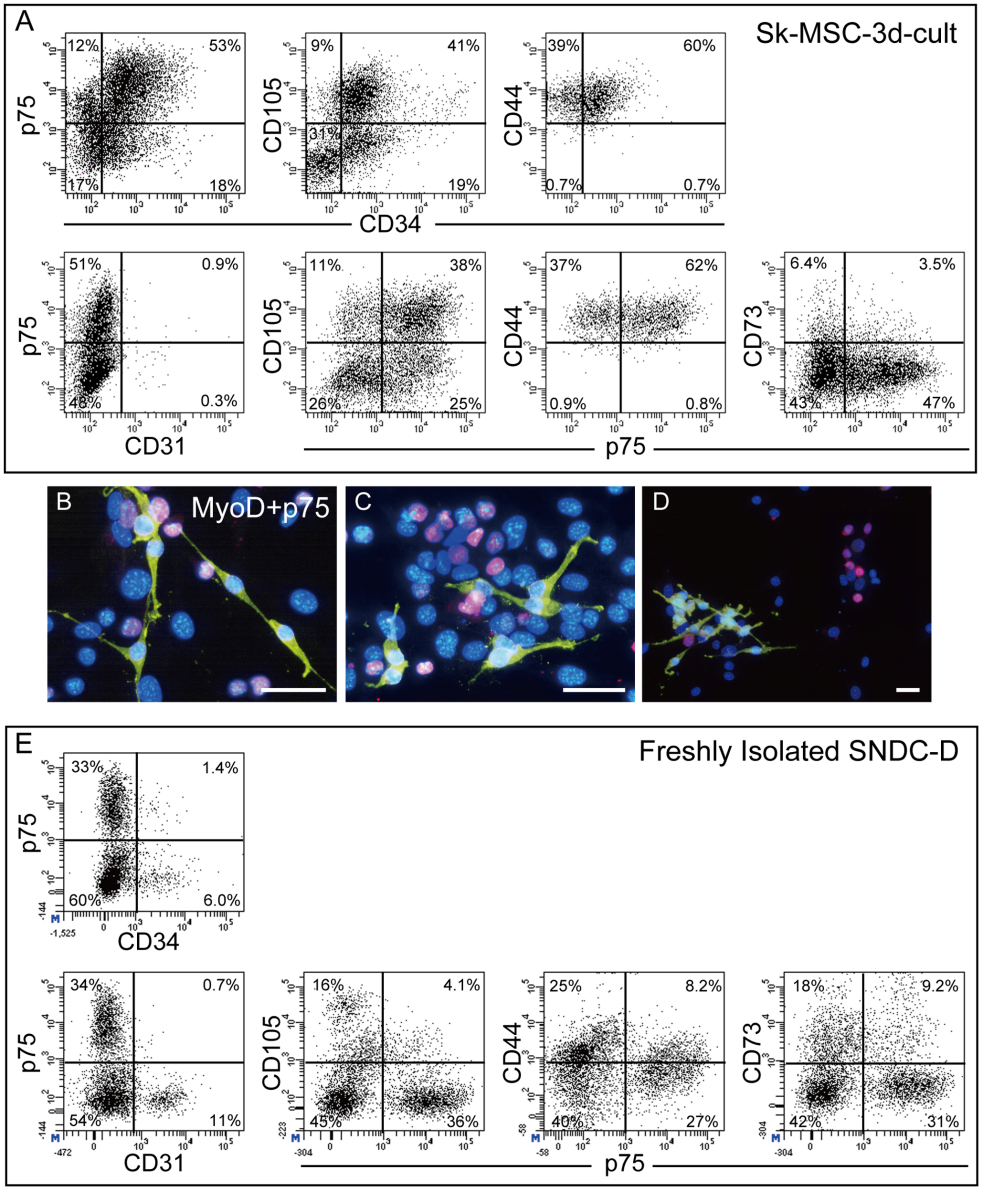
Characterization of Sk-MSC-3d and SNDC-D before transplantation by FACS, and features of p75^+^ cells in Sk-MCS-3d culture. (A) Characteristics of Sk-MSC-3d before transplantation. (B–D) Double-staining of MyoD (pink)- and p75 (yellow)-positive cells in Sk-MSC-3d. Typical bipolar features of p75^+^ cells (immature Schwann cell marker) in the Sk-MSC-3d culture were evident independently of MyoD^+^ cells, and there were no MyoD^+^/p75^+^ cells. Nuclei are stained with DAPI. Scale bars represent 50 µm. (E) Characteristics of freshly isolated SNDC-D.

As described above, Sk-MSCs and SNDCs showed similar differentiation/contribution patterns in damaged nerve niche; therefore, the characteristics of these cells were analyzed using several surface cell markers and FACS (**Fig. 9A and E**). Typical differences were observed in the distributions of cells positive for CD31, CD34 and CD44. Sk-MSC-3d showed fewer CD31^+^ and abundant CD34^+^ cells, and no CD44^−/^p75^+^ cells, as compared with SNDC-D (compare **Fig. 9A and E**). In addition, bipolar p75^+^ cells independent of myogenic cells were observed, thus suggesting the presence of Schwann-progenitor cells in the muscle (**Fig. 9B–D**). However, when Sk-MSC-3d were sorted into 4 fractions as 34^+^/p75^−^, 34^+^/p75^+^, 34^−/^p75^+^ and 34^−/^p75^−^ cells (upper-left of **Fig. 9A**), and were transplanted into the damaged sciatic nerve, the same differentiation/contribution was observed at 4 weeks post-surgery (data not shown). This suggests that Schwann and perineurial/endoneurial progenitor cells are equally distributed in the p75^+^/− and/or CD34^+^/− fractions, as well as vascular-related progenitor cells. Therefore, surface marker characteristics of the Sk-MSC did not show any specific patterns in the present case. In this regard, total cell expansion/transplantation is able to more accurately assess cell number for therapy.

### Therapeutic Capacity of Sk-MSC-7d Vs. Healthy Nerve Grafts for Transected Long Nerve Gap with Acellular Conduit

In order to test the therapeutic efficacy of Sk-MSC for the transected long nerve gap, a 7-mm nerve gap was bridged using an acellular conduit into which Sk-MSC-7d were injected (**Fig. S1A–C**). The healthy nerve graft was also set into a central portion of the conduit as a control experiment (**Fig. S1D–F**).

**Figure 10:**
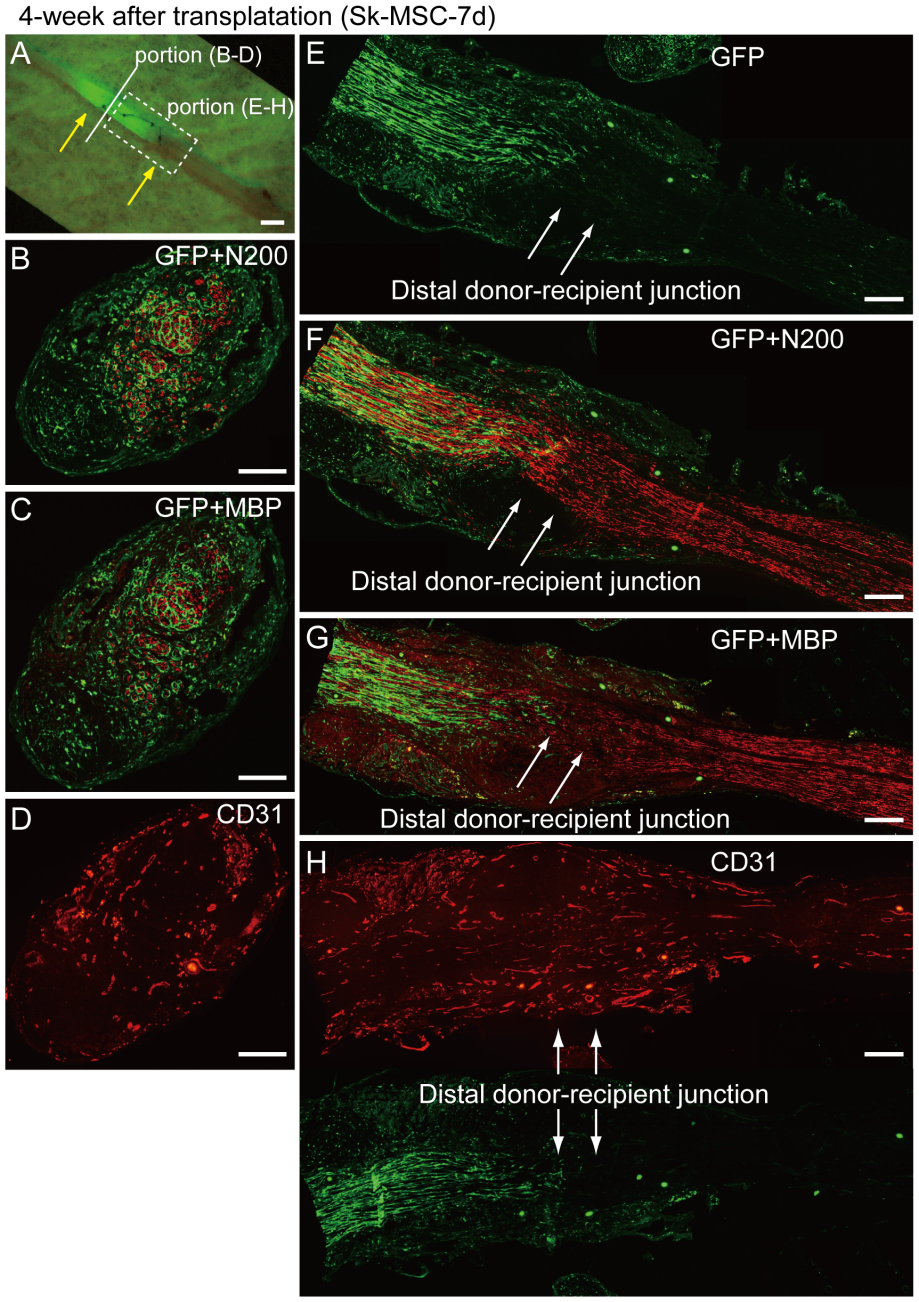
Immunohistochemical analysis in the bridging conduit with Sk-MCS-7d at 4 weeks after surgery. (A) Macroscopic observation of bridging conduit with transplantation. Yellow arrows show both ends of the conduit. Engrafted GFP^+^ donor cells/tissues stretch back to the proximal portion of the recipient nerve. (B–D) Cross-sections obtained from solid line in panel (A). (E–H) Longitudinal sections obtained from dotted square in panel (A). White arrows in (E–H) show the distal donor-recipient junction. A number of N200^+^ axons and MBP^+^ myelin was present, and was mostly encircled by GFP^+^ perineurium (B, C). A large number of blood vessels were also evident (D). These properties were exerted in the distal portion of the conduit, as represented in the longitudinal sections (E–H). Regenerated/extended axons in the conduit already reached the distal donor-recipient junction (arrows in F), thus, axonal continuity was achieved at the 4 week time point. Myelin formation in the junctional portion was likely to be delayed when compared with the axon (arrows in G), but relatively clear reactions could be seen in the downstream recipient nerve portion. Uniform vascular formation through the conduit was also evident (H). Scale bars represent 1 mm (A) and 200 µm (B–H).

After 4 weeks, engrafted GFP^+^ donor cells/tissues stretched back to the proximal portion of the recipient nerve (**Fig. 10A**). In the immunohistochemical analysis, the conduit was filled with GFP^+^ cells/tissues, and counts for N200 (axon), MBP (myelin) and CD31 (blood vessels) in the central portion of conduit were 1847±578 (40% recovery compared to normal intact nerve), 993±413 (31%) and 68±18 (252%, 2.5-fold increase), respectively (n = 4). These values were less than those in normal intact nerve (4625±470, 3179±760 and 27±4; see legend of **Fig. 2**) at this time point. Importantly, the most axons were encircled by GFP^+^ perineurium associated with a 2.5-fold increase in the number of blood vessels in the central portion of the conduit (**Fig. 10B–D**). This trend suggests that axonal elongation and myelination require a precursive perineurial/endoneurial guide and blood vessel supply. These properties were exerted in the distal half of the conduit, as represented in the longitudinal sections (**Fig. 10E–H**), and the regenerated/extended axons in the conduit reached the distal donor-recipient junction (**Fig. 10F**). A delay in myelin formation, as compared to the axon, in the junctional portion was also indicated, whereas uniform vascular formation throughout the conduit could be seen (**Fig. 10G, H**). Thus, a favorable supply of oxygen and nutrition with disposal of waste products could be expected.

**Figure 11:**
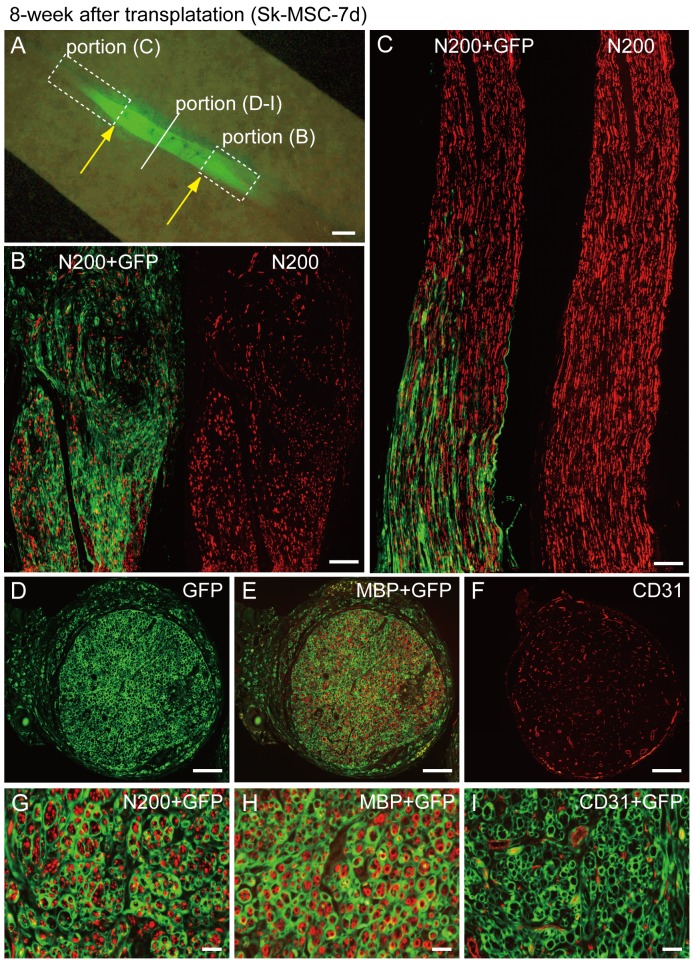
Immunohistochemical analysis in the bridging conduit with Sk-MCS-7d at 8 weeks after surgery. (A) Macroscopic observation of bridging conduit with transplantation. Yellow arrows show both ends of the conduit, and GFP^+^ cells/tissues extended beyond the conduit portion at both the proximal and distal ends. Solid line and dotted squares, which are specified in panel (A) correspond to the panels hereafter. (B, C) Longitudinal sections showed spreading of GFP^+^ cells/tissue beyond the conduit portion and introduction into recipient nerve area. (D–F) Whole cross-sections from the central portion of the conduit. (G–I) High magnification images of staining for N200 (G), MBP (H) and CD31 (I). GFP^+^ perineurium/endoneurium encircled the axons and myelin (G and H), and this is associated with blood vessel formation (F and I), thus showing sufficient recovery of the transected long nerve gap. These properties of engrafted GFP^+^ cells/tissues were similar to those in the former injection experiments into the crash nerve (refer to Fig. 2), showing that Sk-MSCs exert the same differentiation/reconstitution potential for neural/vascular lineages, even in the nerveless bridging conduit. Scale bars represent 1 mm (A), 200 µm (B–F) and 50 µm (G–I).

After 8 weeks, the above properties were further enhanced (**Fig. 11**). Engrafted donor derived GFP^+^ cells showed the exactly same profiles as in the former injection experiment (**Fig. 11A–I** **vs.** **Fig. 3**). Therefore, Sk-MSCs fully exerted the same potential observed in crushed nerve niche, even in the nerveless bridging conduit. In addition, spreading of GFP^+^ cells/tissue beyond the conduit portion and subsequent introduction into the retaining recipient nerve portions on both the proximal and distal sides was observed during expansion from 4 to 8 weeks (**Fig. 10A** vs. **11A**). Therefore, it is likely that donor cell proliferation/differentiation continuously occurred during 4 to 8 weeks after surgery. Mean number of axons, myelin signals and blood vessels in the conduit were N200 = 4328±2026, MBP = 1886±1666 and CD31 = 245±55, which indicated 94%, 60% recovery and 907% (9.1 fold increase) vs. intact nerve, respectively (n = 5).

**Figure 12:**
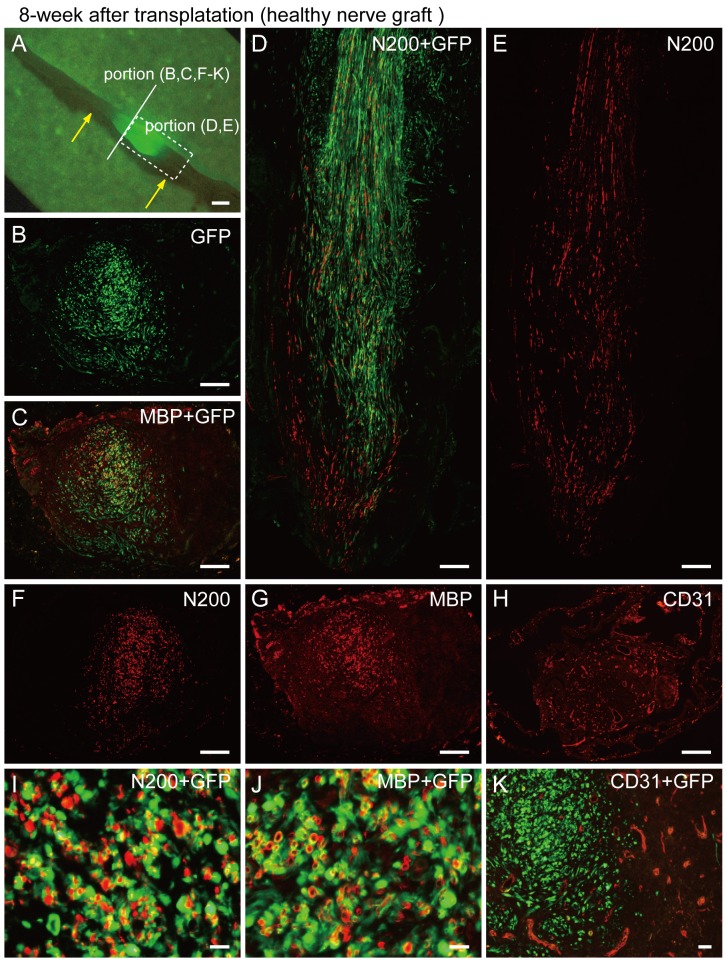
Immunohistochemical analysis in the bridging conduit with healthy nerve graft at 8 weeks after surgery. (A) Macroscopic observation. Yellow arrows show both ends of the conduit. Solid line and dotted squares, which are specified in panel (A), correspond to the panels hereafter. Some GFP^+^ cells/tissues were observed around the central portion of the conduit (A). (B, C, F–K) Cross-sections obtained from the solid line in (A). (D, E) Longitudinal section obtained from dotted square in (A). Interestingly, formation of perineurium/endoneurium was scarcely seen (B, C, I, and J), but a close relationship between GFP circles and N200^+^ reactions (I) and/or double labeling of GFP^+^/MBP^+^ reactions (yellow circle reactions) were frequently observed (J). This indicates that the main contribution of nerve grafts was limited by the supply of Schwann cells. There was no relationship between GFP^+^ cells and CD31^+^ reactions can be seen in panel (K); thus, donor-derived endothelial cells were also unavailable, in contrast to the former injection experiments into the crush nerve. Scale bars represent 1 mm (A), 200 µm (B–H) and 50 µm (I–K).

At 8 weeks after nerve graft transplantation, some GFP^+^ cells/tissues were observed around the central portion of the conduit (**Fig. 12A**), indicating the limited spread of donor cells. Importantly, donor-derived formation of perineurium/endoneurium and blood vessels was scarcely observed except for myelin; thus, the contribution of nerve grafts was limited by Schwann cell supply (**Fig. 12B–K**). Mean numbers of axons, myelin signals and blood vessels in the conduit were N200 = 1663±564 (36% recovery vs. intact nerve), MBP = 824±365 (26% recovery) and CD31 = 145±46 (550%, as 5.5 fold increase, n = 4).

In addition, when surgery was performed using the conduit only (medium+conduit), counts were N200 = 977±771 (21% vs. intact), MBP = 722±585 (23%) and CD31 = 47±14 (177%, 1.7 fold, n = 5). If no conduit was used, the transected nerve gap (7 mm) was scarcely bridged. Therefore, based on the results of nerve graft and medium, the efficacy of the nerve graft was not so large.

**Figure 13:**
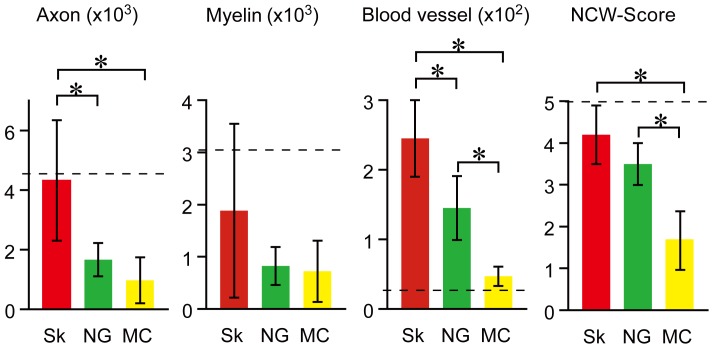
Quantitative data for reconstruction of axon, myelin and blood vessels in bridging conduit and narrow corduroy walking scores at 8 weeks after surgery. Sk-MSC transplantation shows significant and/or favorable quantitative and functional recoveries in all four factors. Dotted lines indicate the mean number of axons, myelin signals and blood vessels in the corresponding portion of normal sciatic nerve (4625±470, 3179±760 and 27±4, respectively). Sk = Sk-MSC-7d, NG = nerve graft and MC = medium control. *P<0.05.

These morphological and quantitative data were clearly representative of in vivo behavioral functions, such as narrow corduroy walking at 8 weeks after complete nerve transection with a 7-mm gap. The narrow corduroy walking scores among three groups were 4.3±0.6 in Sk-MSCs (n = 10), 3.4±0.5 in nerve graft (n = 3) and 1.6±0.6 in media only (n = 5). Representative results are shown as **Movies S1** and **S2** (available at **URL**). As a whole, Sk-MSCs treated mice showed good body balanced locomotion with stable walking (**Movie S1, grade 5**), whereas non-treated (conduit+media) mice tended to limp with every step and had poor balance (**Movie S2, grade 1**). The significance of the above quantitative and functional data for the long nerve gap model at 8 weeks after surgery is summarized in Figure 13.

## Discussion

The first main finding of the present study is that expanded Sk-MSCs preferentially/sufficiently exert their original nerve-blood vessel reconstitution potential, even in damaged peripheral nerve niche with dissolved skeletal myogenic potential. Engrafted donor-derived Sk-MSCs differentiated into all of the main component cells of peripheral nerve support tissues in the severely crushed nerve niche, and enhanced formation of large and small blood vessels was also observed inside/outside the nerve fascicles. Therefore, there were no disadvantages with regard to axonal regrowth resulting from this transplantation. A 10-fold increase in the number of blood vessels appeared in/around the damaged portion when with the intact nerve. This is considered to be a beneficial effect for nerve regeneration, as invasive tissue constructs need to be one step ahead of vascular regeneration in order to ensure adequate delivery of oxygen and nutrients and removal of waste products during regeneration. In addition, the cellular behaviors of engrafted Sk-MSCs were basically the same as those of freshly isolated cells from damaged sciatic nerve (SNDC-D). To the best of our knowledge, stem cells showing suitable multipotency in damaged nerve-specific niche have never been reported. Therefore, it is possible that the Sk-MSCs are the best alternative cell source for peripheral nerve regeneration therapy.

The perineurium plays a very important role as a protective barrier for endoneurial content against the possible noxious influence of external forces and various macromolecules to maintain the neural microenvironment [22], [23]. The perineurium is composed connective tissues, which prevent the passage of large molecules from the epineurium into the nerve fascicle, constituting the “blood-nerve-barrier” system. As expected, donor-derived GFP^+^ perineurium had claudin^+^ tight-junctions, which played a key role in the “blood-nerve-barrier” system, representing functional reconstitution. In nerve fascicles, each individual nerve fiber is further surrounded by the endoneurium. The endoneurium is a thin layer of connective tissue that forms a tubular structure containing the components of nerve fibers such as axons, the myelin sheath and Schwann cells. Thus, the endoneurium is stronger than the axons, and separates and protects them to maintain signal (action potentials) isolation and to establish a delicate communication system within the neuron.

For the above reasons, the second main finding of the present study is that expanded Sk-MSC-7d sufficiently exerts their multiple nerve reconstitution capacity in the bridging acellular conduit for long nerve gaps. In contrast to the nerve crush injury model, inside the bridging conduit is empty space. Nevertheless, transplanted Sk-MSCs developed all peripheral nerve support tissues associated with vigorous induction of blood vessel formation in the conduit. Apparently greater axonal regrowth and myelin formation compared to healthy nerve graft transplantation (about 2.5-hold) was then observed in the conduit, and the transected nerve gap was reconnected within 8 weeks (about 94% recovery vs. intact nerve). The reasons for this difference between Sk-MSCs and nerve graft are thought to be as follows: the nerve graft derived cells showed 1) a lack of contribution to perineurium/endoneurium construction, and blood vessel formation, and 2) a lower migration capacity (see **Fig. 12**). Although nerve graft transplantation showed active contributions to myelin formation, it was insufficient for regenerating the transected nerves. In addition, dedifferentiated Schwann cells in the graft tend to show less mobility in the conduit, because some axonal support can be limitedly observed around the graft portion. Thus, active migration capacity of the Sk-MSCs in and over the conduit (see **Fig. 10** and **11**) also benefits long nerve gap regeneration. According to several reports, perineurial cells are the first cells to reconnect the proximal and distal stumps after the experimental peripheral nerve lesion, and thereby form a guiding structure for re-growing Schwann cells and axons [23]–[27]. It is therefore possible that the capacity to form perineurium/endoneurium is a major advantage of Sk-MSC transplantation vs. other methods. The present results for the number of regenerative axons in the conduit bridging the long nerve gap are far superior to the results for iPS-derived neurospheres+bioabsorbable conduit transplantation (about 4328 at 8 weeks after implantation vs. 200 at 12 weeks) [28].

Synthesis and expression of several neurotrophic, nerve and vascular growth factors by engrafted Sk-MSCs may also play an important supplemental role in nerve regeneration. The present mRNA analysis clearly indicated that Sk-MSC-d7 actively expressed various key factors for axonal re-growth, Schwann cell growth, myelination and total nerve regeneration before and after transplantation (see **Table S1 and** **Fig. 6**). In particular, NGF [29], BDNF [30], GDNF [31], Galectin-1 [32], Ninjurin [33], CNTF [34], LIF [35] and Sox10 [36]–[38] are important for nerve regeneration, while VGEF [39] and HGF [40] are important for blood vessels. Some of these are redundant and/or primarily involved in the nerve regeneration process [41]. Sufficient expression of these factors in Sk-MSC-7d before and after transplantation may induce paracrine effects on recipient cells around transplanted sites and engrafted donor cells as an adjuvant for long gap nerve regeneration. The expression pattern of Sk-MSCs at 17 days after transplantation was similar to that in cells in the damaged nerve (freshly isolated damaged SNDC, see **Fig. 6**).

On autologous cell transplantation, easier access to the cell source is another important issue. In this regard, nestin-expressing hair follicle stem cells were reported as a source to treat injured sciatic nerve [42], [43], and it has also been reported that the same type of stem cells are readily isolated from the human scalp [44]. Significant functional recovery when compared to the non-transplanted group was demonstrated using the severed sciatic nerve model after 2 months of mouse-to-mouse transplantation [43]. However, distance in nerve gap and degrees of morphological and functional recovery rate against the normal mice were unclear; thus, it is difficult to perform direct comparisons with the results of the present study. It is clear that these cells tended to show the limited differentiation into Schwann cells in vivo with fewer perineurium/endoneurium formations, and this is a typical difference in the abilities of the present Sk-MSCs. Therefore, it is likely that hair follicle stem cells have superiority over Sk-MSCs with regard to ease of access. We also believe that severe long gap nerve injury is generally associated with large open scarring of skin and muscles. Thus, donor muscle may be accessible from the injured region.

Finally, the above favorable effects of Sk-MSCs for peripheral nerve regeneration were demonstrated based on the differences between treated and non-treated mice with long nerve gap (good narrow corduroy walking in **Fig. 13** **,** and **Movie S1** vs. **S2**). In addition, whether the above Sk-MSC functions in mice are present in human-Sk-MSCs is the most important issue for translational research. For this purpose, we are currently obtaining the same type of Sk-MSCs from human skeletal muscle. Therefore, this method may be highly practical for human nerve repair therapy.

### Conclusion

The present expanded Sk-MSCs exhibited comprehensive and preferential reconstitution of severely damaged sciatic nerve, following cellular differentiation into all peripheral nerve support cells, such as Schwann cells and perineurial/endoneurial cells, and formed myelin sheath and perineurium/endoneurium, as well as associated blood vessels composed of donor-derived endothelial cells, pericytes and fibroblasts. This represents a potentially useful tool for nerve regeneration therapy. Skeletal muscle is the largest organ in the body, comprising approximately 40–50% of total body mass, and it presumably allows donor cells to be obtained with relative ease and safety. Therefore, Sk-MSCs are potentially a practical source for autologous stem cell therapy for severe nerve injury.

## Materials and Methods

### Animals

Green fluorescent protein transgenic mice (GFP-Tg mice; C57BL/6 TgN[act EGFP]Osb Y01, provided by Dr. M. Okabe, Osaka University, Osaka, Japan) [45] were used as donor mice in cell transplantation (age, 3–8 weeks, n = 80), and wild-type mice (C57BL/6N) were used as recipients (age, 8–12 weeks, n = 210). Cre- and loxP-mice [B6. Cg-Tg (CAG-cre, CZ-M020sc; BRC 01828) and (CAG-floxed Neo-EGFP, REP080sb; BRC 02096)] provided by RIKEN BRC, which is participating in the National Bio-Resource Project of MEXT, Japan (age, 3–8 weeks, n = 10), were used for confirmation studies of cell fusion after transplantation. All experimental procedures were approved by the Tokai University School of Medicine Committee on Animal Care and Use.

### Purification and Preparation of Transplanted Cells

Skeletal muscle-derived multipotent stem cells (Sk-MSCs) were extracted as follows. Whole muscles from the thigh and lower leg (tibialis anterior, extensor digitorum longus, soleus, plantaris, gastrocnemius and quadriceps femoris) of GFP mice (n = 1–2/experiment) were treated with 0.1% collagenase type IA (Sigma-Aldrich, St. Louis, MO) in Dulbecco’s modified Eagle’s medium (DMEM) containing 7.5% fetal calf serum (FCS) with gentle agitation for 2 hours at 37°C and interstitial cells were extracted. Extracted cells were filtered through 70-µm, 40-µm and 20-µm nylon strainers in order to remove muscle fibers and other debris, and were then washed and resuspended in Iscove’s modified Dulbecco’s medium (IMDM) containing 10% FCS, yielding enzymatically extracted cells. The enzymatically extracted mixed cell population (freshly isolated Sk-MSCs), which included both Sk-34 and Sk-DN cell populations, was cultured in 20% FCS/IMDM for 3 or 7 days and achieved about 15–20-fold cell expansion. Therefore, freshly isolated and 3d- and 7d-cultured Sk-MSCs were transplanted for primary studies. On the other hand, in order to confirm whether spontaneous cell fusion occurs, we performed the same transplantation using Cre and loxp mice in the same manner as above.

Whole bone marrow stromal cells (BMSCs) were obtained by flushing the tibias and femurs of GFP mice (n = 2–3/experiment). After elimination of red blood cells, freshly isolated BMSCs were obtained. For the expanded BMSCs, obtained whole marrow cells were cultured in 20%FCS/DMEM for 48 hours, and then floating cells were eliminated, with the remaining adhesive cells being cultured for 5 further days (total of 7 days). Thus, freshly isolated and 7d-cultured BMSCs were transplanted in the same manner as Sk-MSCs (comparative study-I). Medium was changed every 2 days for both cultures. We did not use any cytokines or specific-coating plates for both Sk-MSC and BMSC culture in order to compare their native cell differentiation and tissue reconstructive potential without any modification.

Similarly, freshly isolated sciatic nerve-derived cells (SNDCs) were transplanted (comparative study-II). For this purpose, intact and damaged (4 days after crush injury, see below) sciatic nerves on both sides were excised from GFP mice (n = 5/experiment) and minced, and then SNDCs were extracted enzymatically in the same manner as Sk-MSCs, and were transplanted into damaged sciatic nerves. Note that SNDCs are difficult to culture under the same conditions as Sk-MSCs and BMSCs above, and thus were not studied.

In addition, we performed re-transplantation of re-isolated Sk-MSC-3d into both the damaged skeletal muscle and sciatic nerve models (referred to as the 2^nd^ transplantation) to confirm the changes in original differentiation capacity of Sk-MSCs after the 1^st^ transplantation into damaged nerve niche. For this purpose, after transplanted and engrafted GFP^+^ Sk-MSC-3d were enzymatically re-isolated and sorted by GFP, they were transplanted into damaged skeletal muscle (as described previously [13], [14] and the sciatic nerve. The 2^nd^ transplantation was performed using re-isolated Sk-MSC-3d at 7, 14, and 17 days and at 4 weeks, and re-isolated Sk-MSC-7d at 12 and 17 days after the 1^st^ transplantation.

### Nerve Crush Injury Model and Summary of Transplantation Studies

Under inhalation anesthesia (Isoflurane; Abbot, Osaka, Japan), the right sciatic nerve was exposed through a gluteal muscle incision. The sciatic nerve was then repeatedly crushed with forceps for 7 mm along the longitudinal axis (**Fig. 1A**). Complete nerve crush was confirmed by the appearance of translucent bands across the nerve (**Fig. 1B**). In this case, most of the peripheral nerve support tissues were destroyed, except the epineurium, which is the outermost layer of the nerve (**Fig. 1C**). All transplanted cells were suspended in culture media at a concentration of 1×10^6^ cells/6 µl, and 0.5 µl/nerve was injected into the destroyed hollow portion of the nerve through the remaining epineurium using a fine tip glass pipette. The same amounts of media without cells were also injected as a control.

As a whole, the transplantation study was performed as follows: 1) 3 cases of primary studies using Sk-MSCs (freshly isolated, and 3d- and 7d-cultured); 2) 2 cases of BMSC comparative study-I (freshly isolated and 7d-cultured); 3) 2 cases of SNDC comparative study-II (freshly isolated from non-damaged and damaged nerve); 4) media control comparative study-III; and 5) cell fusion confirmation study using Cre-loxp system. The number of animals used in each experiment is summarized in Table 1.

The nerve crush injury model was also used in the 2^nd^ transplantation experiment (see above), as well as the severe muscle damage model described previously [13], [14].

### Complete Nerve Transection Model with Long Gap (7 mm)

Furthermore, 3 therapeutic transplantation experiments for transected sciatic nerve regeneration were performed using Sk-MSC-7d, intact nerve graft and medium only. For this purpose, the sciatic nerve was transected, and a 7-mm nerve gap was bridged using an acellular conduit made from 70% ethanol dehydrated (3 days) esophageal submucous membrane (mainly longitudinal muscle layer) from wild-type mice. Separation of submucous membrane was performed after dehydration. Then, the bridging conduit was filled by injection of Sk-MSC-7d, and/or medium. Healthy nerve graft (3.5 mm) was obtained from the GFP mouse, and was set in the central portion of the conduit (**Fig. S1**). Surgically treated nerves were analyzed at 4 and 8 weeks after surgery in the same manner as the crush injury model above. Functional recovery was assessed by narrow corduroy walking, and these behaviors were recorded as movies. Behavioral status of surgically treated mice was classified into 5 grades (1 = poor, 2 = fair, 3 = average, 4 = good and 5 = excellent) and averaged as narrow corduroy walking scores. Grade 5 also refers to normal control levels.

### Macroscopic Observation, Immunostaining and Immunoelectron Microscopy

At the end of each transplantation period, recipient mice were given an overdose of pentobarbital (60 mg/kg, i.p.), and engraftment of donor-derived GFP^+^ cells into sciatic nerves was confirmed by fluorescence stereomicroscopy (SZX12; Olympus, Tokyo, Japan; as shown in **Fig. 1F–H**). Mice were then perfused with warm 0.01 M PBS through the left ventricle, followed by fixation with 4% paraformaldehyde/0.1 M phosphate buffer (4% PFA/PB). Nerves (and/or TA muscles, in some cases) were removed and fixed overnight in 4% PFA/PB, washed with graded sucrose (0–25%)/0.01 M PBS series, embedded in optimum-compound (O.C.T compound; Tissue-Tek, Sakura Finetechnical Co., Ltd., Tokyo, Japan) and frozen at −80°C. For muscles, samples were quick frozen in isopentane pre-cooled with liquid nitrogen, and stored at −80°C until use. Subsequently, several 7-µm cross- and longitudinal sections were obtained. Localization of nerve fibers (axons) was detected by rabbit polyclonal anti-Neurofilament 200 (N-200, 1∶1000, room temperature for 1 hour; Sigma, Saint Louis, MO). Myelin formations were detected by rabbit polyclonal anti-myelin basic protein (MBP; 1∶200, room temperature for 2 hours; Millipore, Billerica, MA). Blood vessels were detected using rat anti-mouse CD31 (1∶500, 4°C overnight; BD Pharmingen, San Diego, CA) monoclonal antibody. Immature Schwann cells were detected using rabbit anti-p75 (1∶400, 4°C overnight; CST, Boston, MA) polyclonal antibody, and rabbit anti-claudin-1 (1∶50, 4°C overnight; Zymed, South San Francisco, CA) was used for identification of tight-junctions in the perineurium and endoneurium (blood-nerve-barrier). Myogenic cells were detected with rabbit polyclonal anti-skeletal muscle actin (1∶300, room temperature for 1 hour; Abcam, Cambridge, UK) in histochemistry, and monoclonal anti-MyoD (1∶50, 4°C overnight; Dako, Carpinteria, CA) in culture. Reactions were visualized using Alexa Fluor-594-conjugated goat anti-rabbit and anti-rat antibodies (1∶500, room temperature for 2 hours; Molecular Probes, Eugene, OR). Nuclei were counter-stained with DAPI (4,6-diamino-2-phenylindole).

For immunoelectron microscopy, sections were stained using rabbit anti-GFP antibody (1∶300, 4°C overnight; Molecular Probes) and HRP-conjugated anti-rabbit antibody (1∶200, 4°C overnight; Dako, Carpinteria, CA). Reactions were visualized with DAB after fixation in 1% glutaraldehyde/0.1M phosphate buffer. Visualized sections were then fixed in 1% osmium tetroxide/0.05M phosphate buffer, and were prepared for electron microscopic analysis. Details of immunoelectron microscopy were as described previously [13], [14].

### RT-PCR

In order to test the expression of specific markers for skeletal muscle, peripheral nerve and vascular cell lineages, as well as neurotrophic and vasculogenic factors, bulk cell RT-PCR was performed. Specific primers and analyzed materials are summarized in **Table S1**. Cells were lysed and total RNA was purified using a QIAGEN RNeasy micro kit. First-strand cDNA synthesis was performed with an Invitrogen SuperScript III system using dT30-containing primer (see above), and specific PCR (35 cycles of 30 seconds at 94°C, 30 seconds at 60–65°C and 2 minutes at 72°C) was performed in a 15-µl volume containing Ex-Taq buffer, 0.8 U of ExTaq-HS-polymerase, 0.7 µM specific sense and antisense primers, 0.2 mM dNTPs and 0.5 µl of cDNA. Analysis was performed on Sk-MSCs, BMSCs, SNDCs before transplantation and re-isolated Sk-MSC-3d and -7d (after transplantation). For cells after transplantation, engraftment of GFP^+^ cells into sciatic nerves was first confirmed by fluorescence stereomicroscopy (**Fig. 1F–H**), and nerves were detached at almost 15 mm in length, including the GFP^+^ and GFP^-^ portions. Cells were then extracted by the same procedure as for SNDC preparation, and were sorted into GFP^+^ and GFP^-^ cell fractions using FACSAria (Becton Dickinson Japan, Tokyo). Cells were then analyzed by specific mRNA expression. Analysis was performed for 4–7 samples in each group and for each set of conditions, and relative expression intensity was classified into 3 levels based on housekeeping control gene levels, and were averaged.

### Flow Cytometric Characterization of Sk-MSC-3d and SNDC-D Before Transplantation

Cultured Sk-MSC-3d cells were suspended by trypsin-EDTA (0.05% trypsin, 0.53 mM EDTA; Life Technologies, Tokyo, Japan) and labeled with rat monoclonal anti-mouse CD31 (FITC conjugated, BD or eFluor450 conjugated; eBioscience, San Diego, CA), CD44 (PE-Cy7; BioLegend, San Diego, CA), CD73 (PE, BD or eFluor450; eBioscience), CD105 (PE-Cy7; BioLegend) and CD271 (p75; rabbit polyclonal anti-p75, CST, Boston, MA; and anti-rabbit IgG Dylight 649 or PE, BioLegend, were used as secondary antibodies). Live cells were counted after cells positive for propidium iodide (PI) were excluded as dead cells. All cell analyses and sorting procedure in this study were carried out using FACSAria (Becton Dickinson Japan, Tokyo, Japan). Experiments were repeated three times using 3 mice/experiment in each cell type.

### Quantitative Analysis

Relative engraftment ratio and immunohistochemically detected regenerated axons, myelin and blood vessels in the damaged portion were assessed in each whole cross-section (**Fig. 2**). Relative engraftment ratio was calculated by the percentage of mean GFP^+^ area/total area on whole nerve cross-sections. Regenerated axons, myelin and blood vessels was also determined in whole cross-sections as the number of positive reactions to ant-N-200, -MBP and -CD31, respectively. Reactions were counted using Stereo-investigator (mbf Bioscience, MicroBrightField, Inc. Williston, VT). Analysis was performed in 4–5 sections per sample, and values were averaged. Values are expressed as means ± SD. The narrow corduroy walking score, which was determined for evaluation of functional recovery after transplantation of Sk-MSC-7d, intact nerve graft and medium only in the long nerve gap model were also compared as means ± SD. Differences between groups were tested by parametric Tukey-Kramer post-hoc test. The level of significance accepted a priori was set at p<0.05.

## Supporting Information

Figure S1 **Bridging method for long nerve gap using acellular conduit with Sk-MSC-7d and healthy nerve graft.** Before experiment, esophagus was obtained from wild-type mice, and was immersed/dehydrated in 70% ethanol for 3 days. The serosal ring-shaped muscle and mucosal layer were removed, retaining the submucous membrane mainly composed of longitudinal smooth muscle layer, and this was used as the acellular conduit. (**A–C**) Conduit+Sk-MCS-7d. First, surface of nerve outer membrane was sutured near the proximal and distal ends of the open conduit, and nerves were cut to give a 7-mm distance (**A**). The conduit was then closed with several needles (**B**), and cells were injected into the conduit using a fine glass pipette (**C**). (**D–F**) Conduit+nerve graft. The first step was the same as in (**A** = **D**), and then healthy nerve graft, also obtained from GFP-Tg mouse, was set into the open conduit (**E**), which was then closed (**F**).
(TIF)

Table S1 **Specific primers for RT-PCR.**
(XLSX)

Movie S1 **Typical narrow corduroy walking in conduit+Sk-MSC-7d treated mouse at 8 weeks after complete nerve transection with a 7-mm gap.** This movie includes walking data from 2 mice.
(M2V)

Movie S2 **Typical narrow corduroy walking in conduit+medium treated mouse at 8 weeks after complete nerve transection with a 7-mm gap.** This movie includes walking data from 1 mouse.
(M2V)
