# Supplementary figures and images for: Preferential and Comprehensive Reconstitution of Severely Damaged Sciatic Nerve Using Murine Skeletal Muscle-Derived Multipotent Stem Cells
Source: PLoS One. 2014 Mar 10;9(3):e91257. doi: 10.1371/journal.pone.0091257 (PMC3948784; doi:10.1371/journal.pone.0091257)

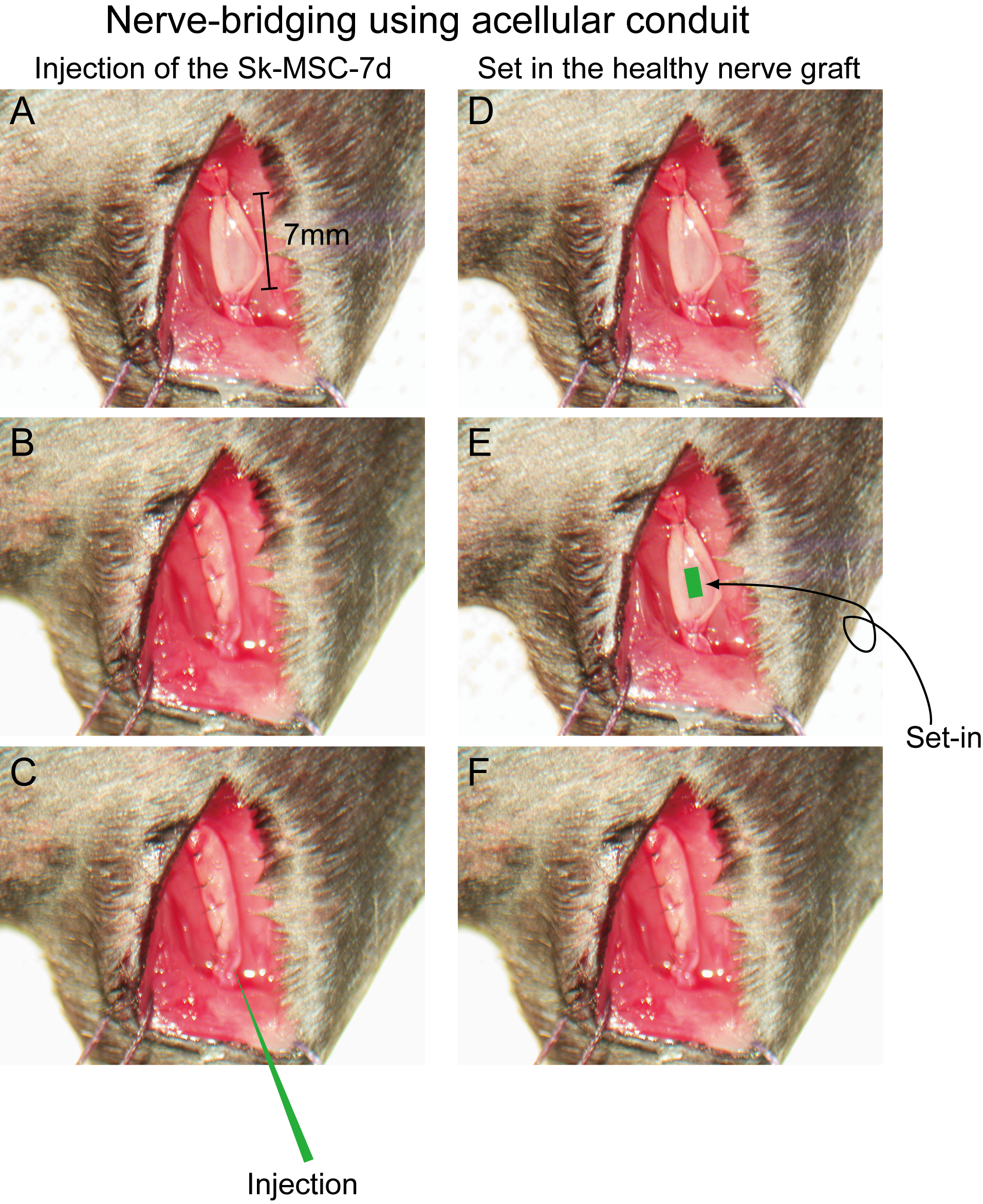

Supplement: Figure S1 — Bridging method for long nerve gap using acellular conduit with Sk-MSC-7d and healthy nerve graft. Before experiment, esophagus was obtained from wild-type mice, and was immersed/dehydrated in 70% ethanol for 3 days. The serosal ring-shaped muscle and mucosal layer were removed, retaining the submucous membrane mainly composed of longitudinal smooth muscle layer, and this was used as the acellular conduit. (A–C) Conduit+Sk-MCS-7d. First, surface of nerve outer membrane was sutured near the proximal and distal ends of the open conduit, and nerves were cut to give a 7-mm distance (A). The conduit was then closed with several needles (B), and cells were injected into the conduit using a fine glass pipette (C). (D–F) Conduit+nerve graft. The first step was the same as in (A = D), and then healthy nerve graft, also obtained from GFP-Tg mouse, was set into the open conduit (E), which was then closed (F). (TIF) [file pone.0091257.s001.tif]
